# Multimorbidity and major adverse cardiovascular events in antipsychotic users: Time-to-event prediction by explainable machine learning

**DOI:** 10.1016/j.isci.2026.115586

**Published:** 2026-04-03

**Authors:** Qi Sun, Wenlong Liu, Cuiling Wei, Yuqi Hu, Lingyue Zhou, Boyan Liu, Rachel Yui Ki Chu, Song Song, Wenxin Tian, Esther Wai Yin Chan, Sherry Kit Wa Chan, Kelvin K.F. Tsoi, Ian Chi Kei Wong, David P.J. Osborn, Daniel Smith, Francisco Tsz Tsun Lai

**Affiliations:** 1 Centre for Safe Medication Practice and Research, Department of Pharmacology and Pharmacy, Li Ka Shing Faculty of Medicine, The University of Hong Kong, Hong Kong, China; 2 Department of Psychiatry, School of Clinical Medicine, Li Ka Shing Faculty of Medicine, The University of Hong Kong, Hong Kong, China; 3 Laboratory of Data Discovery for Health (D24H), Hong Kong Science Park, Sha Tin, Hong Kong, China; 4 Advanced Data Analytics for Medical Science (ADAMS) Limited, Hong Kong, China; 5 Department of Family Medicine and Primary Care, School of Clinical Medicine, Li Ka Shing Faculty of Medicine, The University of Hong Kong, Hong Kong, China; 6 JC School of Public Health and Primary Care, Faculty of Medicine, The Chinese University of Hong Kong, Hong Kong, China; 7 Stanley Ho Big Data Decision Analytics Research Centre, The Chinese University of Hong Kong, Hong Kong, China; 8 Aston Pharmacy School, Aston University Birmingham, Birmingham, UK; 9 Division of Psychiatry, University College London, London, UK; 10 Centre for Clinical Brain Sciences, University of Edinburgh, Edinburgh, UK

**Keywords:** health sciences, medicine, medical specialty, psychiatry, health informatics, internal medicine, public health

## Abstract

Antipsychotic treatment is associated with higher risk of major adverse cardiovascular events (MACEs), and risk may vary by multimorbidity and concomitant medications. Using Hong Kong electronic health records, we followed 26,274 MACE-free adults (18–65 years) with multimorbidity who initiated antipsychotics, capturing demographics, chronic conditions, and prior medication use. We applied a conditional inference survival tree to define clinically interpretable risk profiles and compared ten time-to-event machine learning models using time-dependent ROC, calibration, and decision curve analyses. The highest-risk profile was age >48 years with chronic kidney disease, antibacterial/antiplatelet use, no antidepressant use, and no metastatic cancer (171.3 per 1,000 person-years). A random survival forest model showed the best discrimination (C-statistics 0.841, 0.835, and 0.824 at 1, 3, and 5 years, respectively), with age, antidepressant use, and chronic kidney disease as key predictors. These results support practical cardiovascular risk stratification for antipsychotic initiators with multimorbidity.

## Highlights

• First to map multimorbidity patterns predicting MACE in new antipsychotic users (≤65 years)
• Territory-wide longitudinal EHR enabled robust, population-representative risk stratification
• Predicts both MACE risk and timing, supporting clinically actionable early prevention
• Explainable ML identified key risk drivers and enabled an online interactive risk calculator

## Graphical abstract

**Figure:**
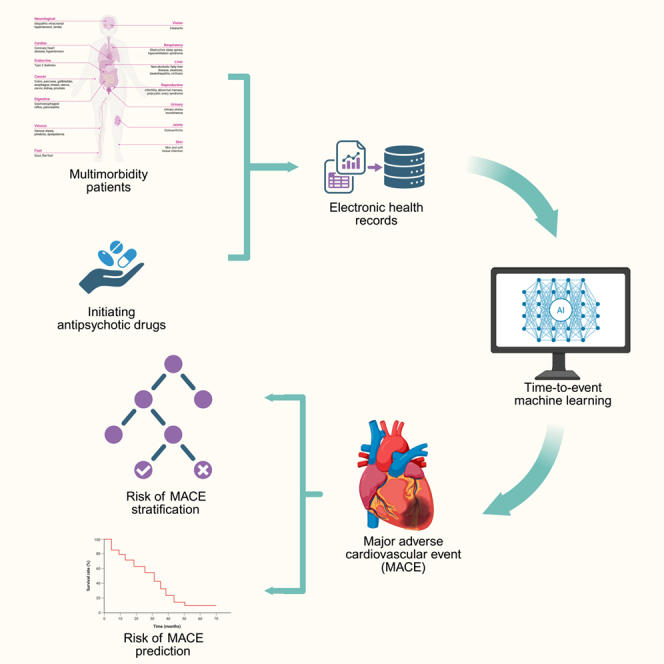

## Introduction

Major adverse cardiovascular events (MACEs), including stroke, acute myocardial infarction (AMI), and cardiovascular-related death (CV death), represent a significant burden for individuals and society.^1^ In 2022, the age-adjusted mortality rate attributable to cardiovascular disease (CVD) in the United States was 224.3 per 100,000.^2^ In addition, it incurs a substantial economic burden on the healthcare system with an estimated annual cost of $503·2 billion from direct and indirect expenses nationwide.^3^ Of particular importance is the population of non-older-adults (aged 65 years or younger), for whom the incidence of early onset CVDs has increased substantially over recent decades, contributing to an increasing share of the total CVD burden and years of life lost.^4^^,^^5^

A disproportionate share of the burden of MACE is borne by people with mental disorders.^6^ In particular, long-term antipsychotic use has been shown to be moderately associated with MACE,^7^ in addition to known adverse effects from behavioral and other prevalent risk factors among populations with mental health problems. Despite a good effectiveness in the treatment of a wide range of psychiatric disorders and symptoms with a generally tolerable safety profile, antipsychotics may be associated with cardiovascular side effects, e.g., QT interval prolongation, hypotension, metabolic side effects, etc.^8^^,^^9^^,^^10^ The mechanisms underlying these associations operate either individually or in combination with other medications.^8^^,^^9^ For instance, Marie et al. reported that Danish older people who used levomepromazine or haloperidol have a 3-fold higher incidence rate of MACE.^7^ Wu et al. observed a similar effect where people using first-generation antipsychotics had 1·7-times higher odds of ventricular arrhythmia and/or sudden cardiac death, and second-generation antipsychotics users had 1·4-times higher such odds.^11^ The current working hypothesis for this consistently observed elevated risk is largely based on the known metabolic abnormalities antipsychotics typically associate with.^12^ A significant proportion of people with severe mental illness also live with multimorbidity, referred to as the co-existence of two or more chronic conditions, which constitutes additional increased risks of MACE.^13^ Ross et al. estimated that there is a 3-fold higher hazard rate (HR) of MACE in rheumatoid arthritis (RA) patients with complex multimorbidity compared to those without it in the UK.^13^ Previous research has also suggested certain specific multimorbidity patterns, such as hypertension-hyperlipidemia, to be more strongly associated with MACE or other adverse health outcomes.^14^ Likewise, it is plausible that there are similar patterns that are predictive of the risk of MACE in antipsychotic users. Nevertheless, such patterns have yet to be investigated with population-representative data.

Given the significant social and economic implications of MACE in antipsychotic users with multimorbidity, it is beneficial to develop a robust, accurate, and interpretable MACE prediction model to inform clinical practices based on large longitudinal databases. Importantly, this will help clinicians prevent or mitigate the risk of MACE in relatively younger patients, e.g., middle-aged adults, on antipsychotics with multimorbidity. In this study, we aim to leverage a territory-wide, population-representative public healthcare database in Hong Kong to characterize the pre-existing multimorbidity profiles of initial antipsychotic users at higher risk of MACE and to develop and validate an explainable machine learning-based prediction model.

## Results

**Figure 1:**
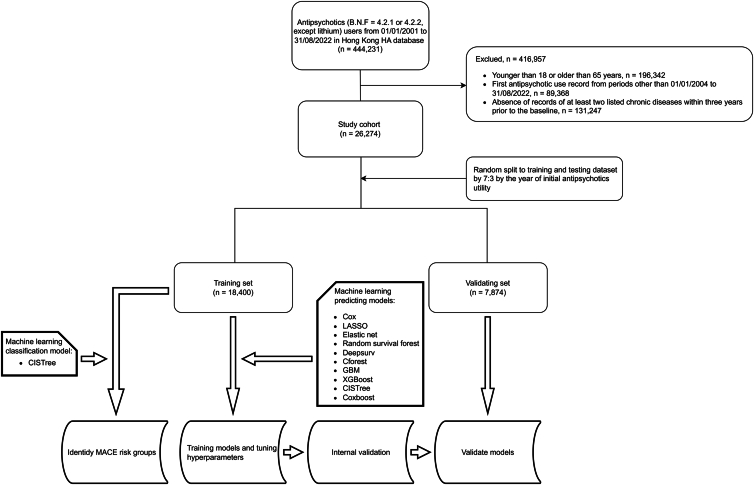
Flowchart of study sample selection and model’s development and validation

In total, 443,231 patients used antipsychotics (B.N.F. 4·2·1 and 4·2·2, except lithium) from January 1, 2004, to August 31, 2022. Following the exclusion of individuals under 18 years of age, those over 65 years of age, non-initial antipsychotic users, and those without multimorbidity, we included a total of 26,274 patients in the study. These patients provided comprehensive data on age, sex, antipsychotic usage, additional medication use, and diagnosed chronic conditions, collected from January 1, 2004, to August 31, 2022, which served as the foundational database for this analysis. After random splitting, 18,400 individuals were allocated to the training set, and 7,874 were in the validation set (Figure 1). Baseline characteristics are tabulated as Table 1. Totally, 1,260 out of 18,400 patients experienced MACE within a median follow-up period of 514 (interquartile range [IQR] 33 to 2,850) days in the training cohort, and 524 out of 7,874 people in the validation cohort experienced MACE during a median follow-up time of 554 (IQR: 36 to 2,878) days. The MACE incidence rate (IR) in the training set was 16·153 (95% confidence interval (CI): [15·273, 17·070]) per 1,000 person-years.

**Table 1:** Baseline characteristics

	Training cohort	Validating cohort
N.	18,400	7,874
Sex (%)		
Male	9,387 (51·0)	4,126 (52·4)
Female	9,013 (49·0)	3,748 (47·6)
Age (median [IQR])	55 [47,60]	55 [47,61]
18–30 (No. [%])	885 (4·8)	385 (4·9)
31–45 (No. [%])	3,223 (17·5)	1,361 (17·3)
46–65 (No. [%])	14,292 (77·7)	6,128 (77·8)

**Multimorbidity (%)**		

Alcohol misuse	1,111 (6·0)	555 (7·0)
Asthma	769 (4·2)	336 (4·3)
Atrial fibrillation	301 (1·6)	162 (2·1)
Cancer lymphoma	228 (1·2)	111 (1·4)
Cancer metastatic	6,912 (37·6)	2,863 (36·4)
Cancer non-metastatic	5,812 (31·6)	2,441 (31·0)
Chronic kidney disease	1,713 (9·3)	752 (9·6)
Chronic pain	5,755 (31·3)	2,466 (31·3)
Chronic pulmonary disease	1,179 (6·4)	514 (6·5)
Chronic viral hepatitis B	678 (3·7)	290 (3·7)
Cirrhosis	1,224 (6·7)	509 (6·5)
Dementia	197 (1·1)	76 (1·0)
Depression	5,597 (30·4)	2,348 (29·8)
Diabetes	3,196 (17·4)	1,396 (17·7)
Epilepsy	425 (2·3)	212 (2·7)
Hypertension	4,969 (27·0)	2,171 (27·6)
Hypothyroidism	462 (2·5)	177 (2·2)
Inflammatory bowel disease	6 (0·0)	1 (0·0)
Irritable bowel syndrome	137 (0·7)	43 (0·5)
Multiple sclerosis	54 (0·3)	35 (0·4)
Parkinson disease	91 (0·5)	38 (0·5)
Peptic ulcer disease	411 (2·2)	190 (2·4)
Peripheral vascular disease	20 (0·1)	13 (0·2)
Psoriasis	90 (0·5)	35 (0·4)
Rheumatoid arthritis	192 (1·0)	80 (1·0)
Schizophrenia	651 (3·5)	271 (3·4)
Severe constipation	2,827 (15·4)	1,235 (15·7)
Retinal vascular occlusion	53 (0·3)	18 (0·2)

**One-year medication history (%)**		

Antibacterial drugs	10,120 (55·0)	4,302 (54·6)
Drug used in diabetes	3,386 (18·4)	1,484 (18·8)
Antidepressant drugs	7,228 (39·3)	3,095 (39·3)
Corticosteroids	6,411 (34·8)	2,707 (34·4)
Antiviral drugs	1,833 (10·0)	736 (9·3)
β-adrenoceptor blocking drugs	3,343 (18·2)	1,445 (18·4)
Antiplatelet drugs	1,312 (7·1)	547 (6·9)
Anti-arrhythmic drugs	570 (3·1)	264 (3·4)
Antianginal drugs	4,739 (25·8)	2,073 (26·3)
Drugs used in hypertension and heart failure	2,569 (14·0)	1,136 (14·4)
Diuretics	3,941 (21·4)	1,681 (21·3)
Lipid regulating drugs	2,329 (12·7)	1,036 (13·2)
Anticoagulants and protamine	364 (2·0)	137 (1·7)
Drugs affecting the immune response	286 (1·6)	131 (1·7)
Immunoglobulins	1 (0·0)	1 (0·0)
Cough preparations	1 (0·0)	0 (0·0)
Cytotoxic drugs	0 (0·0)	1 (0·0)

**Antipsychotic agent initiated (%)**		

Amisulpride	118 (0·6)	52 (0·7)
Aripiprazole	247 (1·3)	105 (1·3)
Asenapine	0 (0·0)	0 (0·0)
Brexpiprazole	14 (0·1)	8 (0·1)
Chlorpromazine	1,897 (10·3)	834 (10·6)
Clozapine	10 (0·1)	7 (0·1)
Flupenthixol	182 (1·0)	88 (1·1)
Fluphenazine	0 (0·0)	0 (0·0)
Haloperidol	9,862 (53·6)	4,235 (53·8)
Lurasidone	6 (0·0)	3 (0·0)
Molindone	0 (0·0)	0 (0·0)
Olanzapine	355 (1·9)	142 (1·8)
Paliperidone	14 (0·1)	5 (0·1)
Pericyazine	21 (0·1)	10 (0·1)
Perphenazine	112 (0·6)	41 (0·5)
Pimozide	2 (0·0)	1 (0·0)
Quetiapine	3,370 (18·3)	1,435 (18·2)
Risperidone	1,243 (6·8)	542 (6·9)
Sertindole	2 (0·0)	0 (0·0)
Sulpiride	785 (4·3)	346 (4·4)
Thioridazine	28 (0·2)	8 (0·1)
Thiothixene	0 (0·0)	1 (0·0)
Trifluoperazine	377 (2·0)	154 (2·0)
Ziprasidone	16 (0·1)	1 (0·0)
Zuclopenthixol	13 (0·1)	6 (0·1)

**Outcomes distribution**		

MACE cases (%)	1,260 (6·8)	524 (6·7)
Time to MACE ∗(days, median [IQR])	514 [33, 2,850]	554 [36, 2878]
MI cases (%)	317 (1.7)	128 (1.6)
Time to MI (days, median [IQR])	3,266 [1,486, 4,805]	3,251 [1,479, 4,827]
Stroke cases (%)	806 (4.4)	338 (4.3)
Time to Stroke (days, median [IQR])	3,186 [1,420, 4,746]	3,188 [1,424, 4,753]
CV-death (%)	309 (1.7)	130 (1.7)
Time to CV-death (days, median [IQR])	3,249 [1,477, 4,794]	3,241 [1,470, 4,804]

Note: ∗: the timing of MACE in cases where MACE occurred on the initial day of antipsychotic use was recorded as 0·5 days.

### Identification of MACE risk group

**Figure 2:**
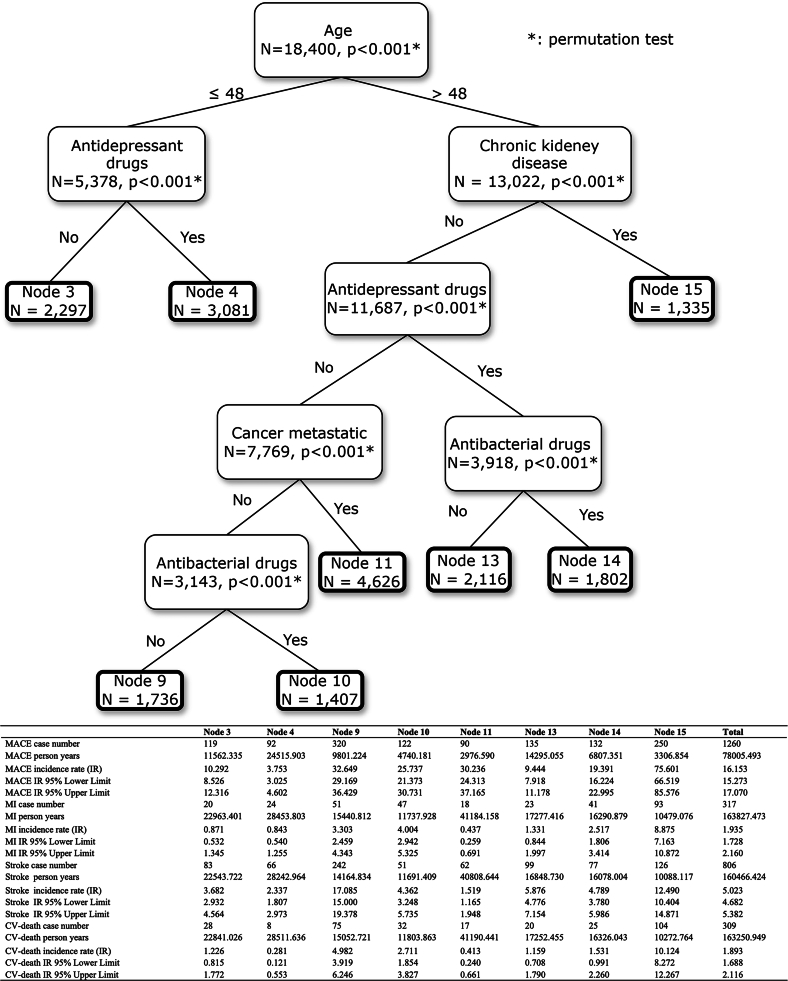
Conditional inference survival tree Data are represented as point estimated incidence rates and 95% confidence interval.

There were 18,400 patients ranging across the 30 nodes generated by CISTree algorithm. In the Figure 2, node 55, which represents patients older than 48 years, living with chronic kidney disease (CKD), having used antibacterial and antiplatelet drugs but not antidepressants, and without metastatic cancer, has the highest IR, 171·317 (95% CI: [130·088, 221·467]) per 1,000 person-years. In node 57, people older than 48 years, diagnosed with CKD and metastatic cancer have the lowest MACE risk across all nodes, i.e., 0·000 (95% CI: [0·000, 32·939]) per 1,000 person-years. The CISTree suggests that age, antidepressant drugs, and CKD are the top three factors most related to MACE status. In sensitivity analyses, age and CKD consistently show high relevance to MACE.

### Time-to-MACE survival prediction

A total of 24 variables were chosen for modeling. These covariates include age, atrial fibrillation, metastatic cancer, non-metastatic cancer, CKD, cirrhosis, diabetes, hypertension, schizophrenia, severe constipation, antidepressant drugs, corticosteroids, antibacterial drugs, antiviral drugs, drugs used in diabetes, β adrenoceptor blocking drugs, antiplatelet drugs, antianginal drugs, drugs used in hypertension and heart failure, diuretics, lipid regulating drugs, haloperidol, quetiapine, and risperidone.

### Internal validation diagnostics

The hyperparameters tuning results and the chosen values are in Table S3. Table S4 presents the survival C index, survival calibration score, and right-censored log loss for each tuned machine learning model in training set. RSF and XGBoost demonstrate better overall internal performance among the ten models.

### Validation using the validating cohort

**Figure 3:**
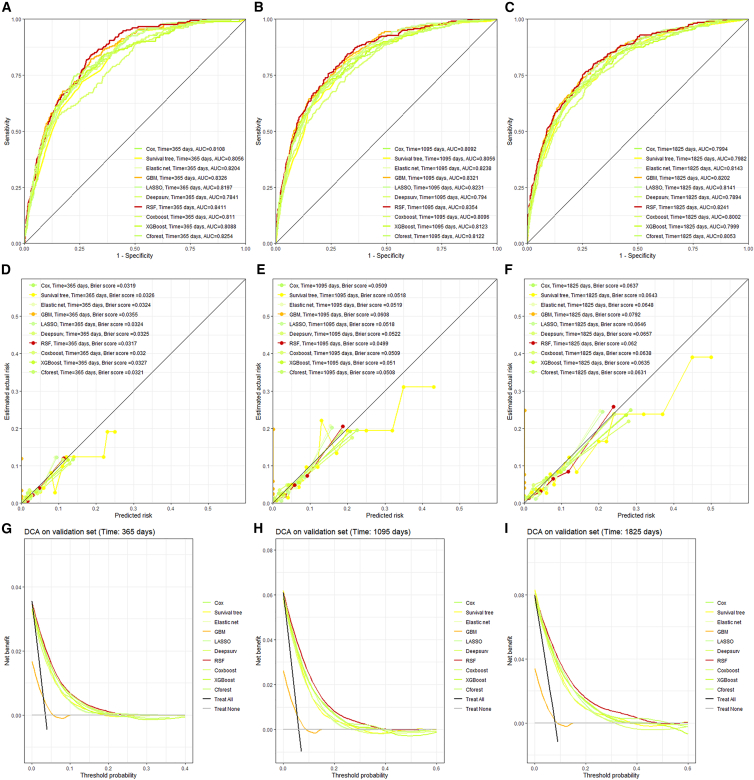
ROC, Calibration, and DCA plot (A–C) Are three ROC plots at the 1st, 3rd, and 5th year after the initial date of antipsychotics use. (D–F) Are three calibration plots at different time. (G–I) Are DCA plots, where the odds of the threshold probability shows the ratio of the benefit achieved by curing a true positive case to the harm caused by unnecessary treatment to a false positive case, so the threshold should be determined by clinicians.

We compared the discrimination capacity of ten tuned machine learning methods on the validation dataset.^15^ The time-dependent ROC plots are presented in Figures 3A–3C. According to these figures, RSF performs the best among the three time points with an AUC from 0·824 to 0·841, while Deepsurv performs the worst with an AUC below 0·800.

The calibration performance of each model was gauged and visualized using survival Brier scores and calibration plot, in Figures 3D–3F, respectively. These figures show that RSF has the best calibration ability since their calibration curves align with the diagonals better than other models, and its brier score is relatively smaller, at the three time points.

Comparing the decision curve analysis (DCA) curves among the ten models is necessary (Figures 3G–3I).^16^ The higher the net benefit under certain threshold probability, which was used as the threshold to predict MACE experience, the better the model performs in practice. Here, RSF consistently outperforms compared to other models (in case of avoiding missed diagnosed case, lowering false negative, the net proportion of true negative can serve as the net benefit, in Figure S1, where RSF performs best. The net benefit formula is provided in Methods S1).^16^^,^^17^

Across the three dimensions of model evaluation—discrimination, calibration, and clinical utility—in the validation set, and in sensitivity analyses that included all prior diagnoses and excluded haloperidol users, the RSF model demonstrated the most comprehensive performance (higher survival C index, lower survival Brier score, and greater net benefit).

### Machine learning model interpretation

**Figure 4:**
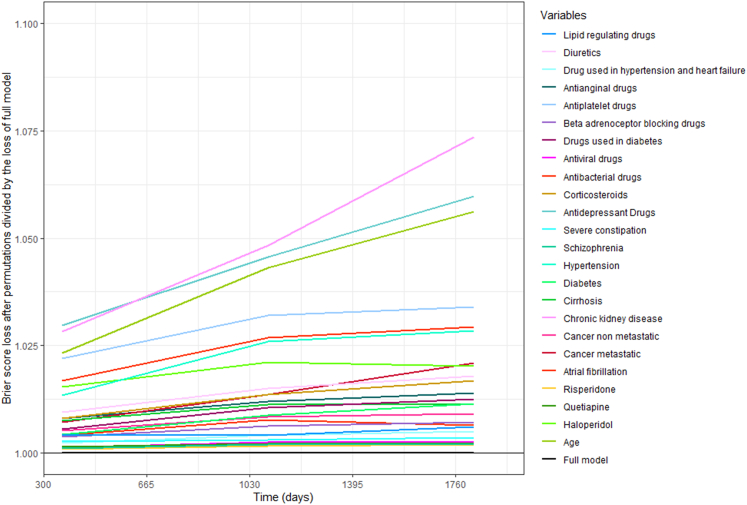
Time-dependent feature importance plot The higher the brier score loss shown on *y* axis, the more importance the factor owns.

We interpreted the RSF model by identifying variable importance to discover the most important predictors over time. Figure 4 demonstrates how the importance of different predictors varied over time, suggesting that age, CKD, and antidepressant drugs are the top three predictors. Furthermore, for predicting MACE status in the first year, antidepressant drug is the most important predictor, which is surpassed by CKD in the third and fifth years. Analogizing important predictors from RSF model to nodes of CISTree, all factors appearing in the CISTree model are included in RSF. Particularly, the top three factors of CISTree, age, CKD, and antidepressant drugs, are not only strongly associated with the time-to-event outcome of MACE, but also have significant prediction/risk classification value.

**Figure 5:**
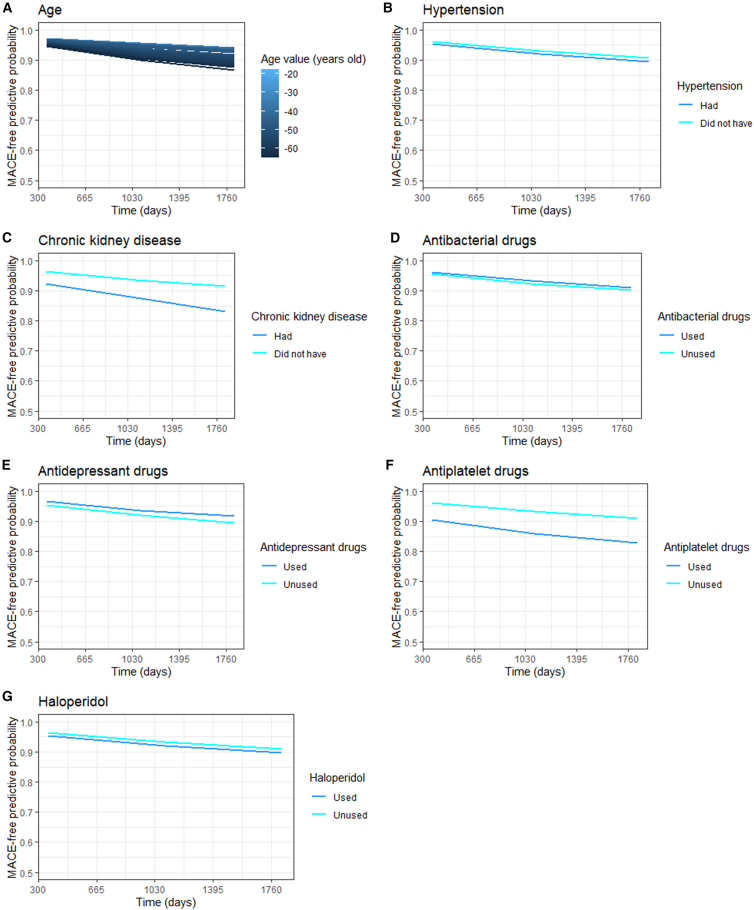
Top seven features' time-dependent partial dependence plot (A) Age. (B) Hypertension. (C) Chronic kidney disease. (D) Antibacterial drugs. (E) Antidepressant drugs. (F) Antiplatelet drugs. (G) Haloperidol. Data are represented as mean MACE-free predictive probability.

Additionally, Figure 5, the partial dependent plot (PDP), reveals how the top seven prediction-important features (the rest variables PDP are in Figure S2) values affects MACE-free predictive probability when other factors remain constant. It shows that with increasing age (Figure 5A), a patient’s MACE status worsens, while other predictors are held constant. Similar phenomena exist with the usage of antiplatelet drugs (Figure 5F), haloperidol (Figure 5G), and a CKD diagnosis (Figure 5C). The difference in MACE-free predictive probability between population non-recorded and recorded CKD is 3·9% on the first follow-up year and 8·0% on the fifth year; the difference in MACE-free predictive probability between patients unused and used antiplatelet drug is 5·7% and 8·3% on the first and fifth year respectively. In contrast, antidepressant use is associated with a 1·5% higher MACE-free probability.

**Figure 6:**
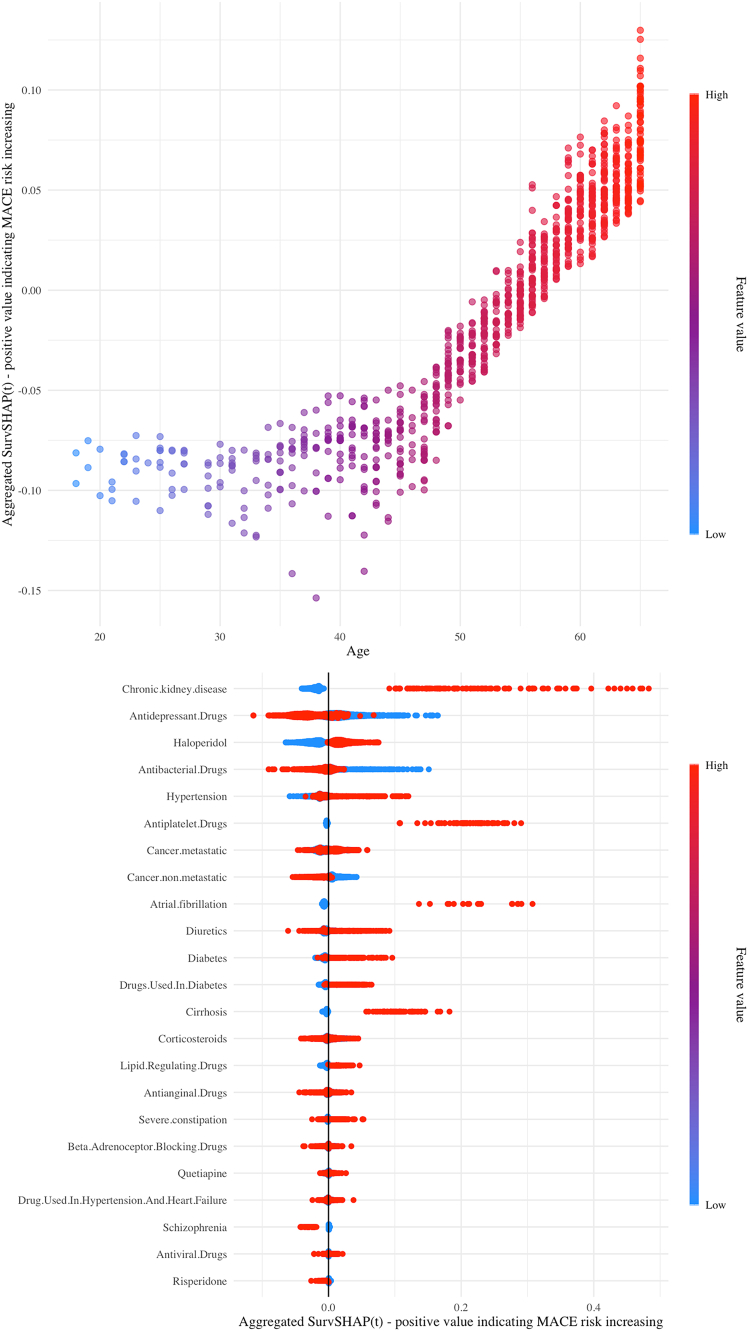
Global SHAP plot for age and other factors A positive SHAP value indicates that the feature value of certain sample has an increasing effect on the prediction value of MACE risk against the average predictive MACE probability in whole population. The color of the dots symbolizes the numerical value of the variables.

The global SHAP plot (Figure 6) illustrates how feature values impact the predicted risk of MACE compared to the average predicted probability. Older age is associated with a higher estimated MACE risk, while younger age (<43 years old) indicates a lower and consistent MACE risk. Additionally, CKD and haloperidol use are linked to an increased estimated MACE risk (an SHAP plot for individual explanations is shown in Figure S3).

Based on the results from RSF model, we built a pilot online interactive platform, HKU Antipsychotics Multimorbidity MACE calculator (Figure S4, HKUAPMMCal, https://hkupmmcal.shinyapps.io/HKUAPMMCal) to potentially aid clinical practice.

## Discussion

We found that initial antipsychotic users with multimorbidity, aged 48 and older, living with CKD, using antibacterial and antiplatelet medications (but not antidepressants), and without metastatic cancer, has the highest risk of MACE, followed by some other specifically characterized groups of antipsychotic users. Among various machine learning models evaluated for discrimination, calibration, and clinical utility, the RSF model outperformed the others, demonstrating the highest validation performance and potentially offering the greatest net benefit in aiding clinical management. Older age, CKD, and the absence of antidepressant use emerged as the top three crucial predictors, with older age and CKD indicating a substantially heightened risk of MACE. Additionally, use of haloperidol or antiplatelet drugs was associated with a significantly increased incidence of MACE in this population.

Consistent with our observation on specific predictors as interpreted from the model, a US cohort study reported a slight increase in mortality risk within seven days among patients with AMI starting haloperidol compared to other typical antipsychotics (hazard ratio [HR]: 1·50, 95% CI 1·14 to 1·96).^18^ Similarly, Mikkel et al. found that low-dose quetiapine (≤50 mg tablets prescribed for over 365 days) was associated with a 1·13 times higher risk of MACE in their intention-to-treat analysis (95% CI: [1·02, 1·24]) and a 1·52 times higher risk in as-treated analysis (95% CI: [1·35, 1·70]).^19^ These findings are consistent with our conclusions as shown in the PDP (Figures 5G and 2A). A cohort study in Wales demonstrated an increased risk of MACE among individuals with CKD (eGFR 30 mL/min/1·73m^2^): HR 3·43 (95% CI: [3·22, 3·64]) in the heart failure and atrial fibrillation multimorbidity cluster, and HR 4·18 (95% CI: [3·65, 4·78]) in the heart failure, peripheral vascular disease, and diabetes cluster, as observed in another cohort study in Sweden.^20^ Briana et al. also noted that among patients with type 2 diabetes, those with at least four co-morbidities had a 2·68 times higher risk of cardiovascular death (95% CI: [2·52, 2·85]) compared to those without multimorbidity, 19 years after the diagnosis of type 2 diabetes.^21^

We developed and evaluated machine-learning models to predict MACE risk among incident antipsychotic users with pre-existing multimorbidity, with a comprehensive comparative assessment across models. Machine learning algorithms are renowned for their flexibility and comprehensiveness in considering all available information specific to individuals. In clinical practice, it is particularly valuable for clinicians and health consultants to assess not only the likelihood of a patient experiencing MACE but also the approximate timing. This allows for more precise preventive measures, potentially conserving medical resources and alleviating the healthcare burden on communities and nations.

Previous machine learning studies on MACE prediction have primarily focused on predicting whether MACE will occur within a certain period of time. For instance, Ayako et al. used a random forest model with MRI data to predict MACE among adults with repaired tetralogy of Fallot, achieving a C-index of 0·82 (95% CI: [0·74, 0·89]).^22^ Similarly, Jain et al. employed gradient boosting machines (GBMs) and XGBoost algorithms to predict MACE occurrence following orthotopic liver transplantation, achieving a C index of 0·71 (95% CI: [0·63, 0·79]).^23^ These studies, however, are limited in clinical applicability without sufficiently assessing the trade-offs between sensitivity, specificity, as well as burdens from false positives and false negatives, which we thoroughly investigated in a series of decision curve analyses. And they did not demonstrate a thoughtful selection process of the appropriate model through comparison by a wide range of diagnostics.

More importantly, this territory-wide database enhances the robustness and prediction performance of the RSF model due to its large size, representativeness, and continuous updates. The selected predictors are readily accessible compared to more complex data sources like highly specific laboratory test results, MRI, or ultrasound scans, as all variables can be obtained through routine clinical assessments and patient records. This accessibility facilitates the swift computation of predicted risk for individual patients during consultations, enabling clinicians to easily explain risk prospects. Therefore, our study is motivated by clinical needs, reproducible, and applicable in clinical practice.

### Conclusion

This study identified a high-risk group for MACE among non-older patients with multimorbidity receiving antipsychotics and established a validated machine learning time-to-event MACE prediction model. Further external validation is necessary to assess the model’s applicability across different settings. Additionally, future research should investigate potential causal relationships between the identified factors and MACE risk, as inferring causality is beyond the scope of this study.

### Limitations of the study

It is important to note that despite its plausible local applicability and clinical value. Multiple independent external validation studies are essential to assess the model’s generalizability across different populations and settings, should this prediction model prove to be needed elsewhere. Thus, we provided an external validation proposal by using JMDC data in Methods S2.^24^ Additionally, the observed associations between antipsychotics, concurrent medications, multimorbidity patterns, and MACE risk are not causal before comprehensive study. For example, confounding by indication likely underlies the association between use of antiplatelet agents and a higher risk of MACE: in real-world data, antiplatelet use often signals higher baseline cardiovascular risk (e.g., prior coronary artery disease, PCI/MI history).^25^ Consequently, higher crude event rates are frequently observed in nonrandomized data. Other associations are biologically plausible: users of antidepressants showed a lower incidence of MACE, potentially reflecting the benefits of effectively treating depression, an independent cardiovascular risk factor.^26^^,^^27^^,^^28^ Conversely, haloperidol use and chronic kidney disease are associated with higher MACE risk, consistent with QT-interval prolongation and the increased cardiovascular risk accompanying reduced glomerular filtration rate, respectively.^29^ Nevertheless, given the predictive and observational nature of our study, these associations warrant further investigation. Methods such as inverse probability weighting or instrumental-variable analyses, applied within cohort or target trial emulation designs, may help clarify causal relationships.

## Resource availability

### Lead contact

Requests for further information and resources should be directed to and will be fulfilled by the lead contact, Francisco Tsz Tsun Lai, (fttlai@hku.hk).

### Materials availability

This study was conducted entirely based on electronic health records and no biological agents or animal models were used throughout the entire study. The web-based application generated in this study will be made available on request, but we may require a payment and/or a completed copyright transfer agreement if there is potential for commercial application.

### Data and code availability

• The Hong Kong Hospital Authority (HA) data reported in this study cannot be deposited in a public repository because this real-world health data governed by strict security and privacy requirements. To request access, contact the Hong Kong Hospital Authority via (enquiry@ha.org.hk).
• All original code has been deposited at Zenodo (https://zenodo.org/records/18816892) and is publicly available at https://doi.org/10.5281/zenodo.18816892 as of the date of publication.
• Any additional information required to reanalyze the data reported in this study is available from the lead contact upon request.

## Acknowledgments

The authors thank the Hospital Authority for the generous provision of data. The 10.13039/501100005847Health and Medical Research Fund funded this study (reference number: 19201271). Informed consent was not required, as the database was anonymized, and the data collection process complied with data privacy regulations.

## Author contributions

Q.S. and F.T.T.L. contributed the conception of this work. The study was designed by all authors. Q.S. contributed to the acquisition and analysis of the data, and all authors interpreted the data. Q.S. and F.T.T.L. drafted the manuscript. Every contributor critically reviewed the manuscript for essential intellectual content, endorsed the final version for publication, and accepted responsibility for all components of the work. Q.S. is the first author and F.T.T.L. is the corresponding author. F.T.T.L. had full access to all the data in this work and all authors took ultimate responsibility for the decision to submit this manuscript for publication.

## Declaration of interests

The authors declare that there is no conflict of interest.

## STAR★Methods

### Key resources table

**Table:** 

REAGENT or RESOURCE	SOURCE	IDENTIFIER
**Deposited data**		

We used territory-wide electronic health records spanning from January 1, 2001, to August 31, 2022, maintained by the Hong Kong Hospital Authority (HA). The HA oversees all local public hospitals and the majority of public outpatient clinics, providing health services for over 7.5 million Hong Kong residents. No new datasets of a standardized datatype (e.g., sequencing, proteomics, crystallography) or microarray, proteomics, or RNA-seq data used in whole analysis.	Hong Kong Hospital Authority (HA)	1. Related publication: Gao, L., Leung, M. T. Y., Li, X., Chui, C. S. L., Wong, R. S. M., Au Yeung, S. L., Chan, E. W. W., Chan, A. Y. L., Chan, E. W., Wong, W. H. S., Lee, T. M. C., Rao, N., Wing, Y. K., Lum, T. Y. S., Leung, G. M., Ip, P., & Wong, I. C. K. (2021). Linking cohort-based data with electronic health records: a proof-of-concept methodological study in Hong Kong. *BMJ open*, *11*(6), e045868. https://doi.org/10.1136/bmjopen-2020-045868 2. HA data sharing portal website: https://www3.ha.org.hk/data 3. HA website: https://www.ha.org.hk/visitor/ha_index.asp

**Software and algorithms**		

We conducted all analyses using *R* (version 4·4·0), employing the “*partykit*” (version 4·4·0) package to establish the CISTree model, “*Boruta*” (version 9.0.0) for variable selection, “*mlr3proba*” (version 0.8.6) to build ten machine learning models, “*survex*” (version 1.2.0) for model explanations, and the “*shiny*” (version 1.12.1) package to construct the prediction platform.	R is a free software environment for statistical computing and graphics. (please check: https://www.r-project.org/)	*R* (version 4·4·0); “*partykit*” (version 4·4·0), “*Boruta*” (version 9.0.0), “*mlr3proba*” (version 0.8.6), “*survex*” (version 1.2.0), and “*shiny*” (version 1.12.1)
Analysis code for this study	Zenodo	https://zenodo.org/records/18816892; DOI: https://doi.org/10.5281/zenodo.18816892

### Experimental model and study participant details

This study used human participants only, via routinely collected electronic health records (EHRs) from the Hong Kong Hospital Authority (HA). No animals, plants, microbes, cell lines, primary cell cultures, or other experimental models were used; therefore, items related to genotype/strain, cell line authentication, and mycoplasma testing are not applicable.

We identified a territory-wide cohort from Hong Kong HA EHRs (2001–2022) comprising initial antipsychotic initiators (from 2004 onward; BNF 4.2.1/4.2.2, lithium excluded) aged 18–65 years who had multimorbidity (≥2 chronic conditions recorded within 3 years before the index date) and an available 1-year pre-index medication history. We excluded individuals with prior MACE or invalid/missing records, and followed patients from initiation to MACE, death, or end of data (31 Aug 2022), with 2001–2003 as a washout period to define initial use. Finally, a total of 443,231 patients used antipsychotics between Jan 1, 2004 and Aug 31, 2022. After exclusion, 26,274 eligible patients remained. These were randomly split into a training cohort (n = 18,400) and a validation cohort (n = 7,874). During follow-up, 1,260/18,400 patients in the training cohort and 524/7,874 in the validation cohort experienced MACE.

In this study, the most influential predictors identified for MACE risk prediction were age, chronic kidney disease, and antidepressant use; sex did not emerge as a top predictor in the primary interpretability outputs reported.

### Method details

#### Ethics considerations

The Institutional Review Board of the University of Hong Kong/Hospital Authority Hong Kong West Cluster (HKU/HA HKW IRB; reference no. CIRB-2022-015-5) approved this study. The requirement for informed consent was waived because the study used only anonymised electronic health records.

#### Study design and data source

This retrospective machine learning cohort study utilized territory-wide electronic health records spanning from January 1, 2001, to August 31, 2022, maintained by the Hong Kong Hospital Authority (HA). The HA oversees all local public hospitals and the majority of public outpatient clinics, providing health services for over 7.5 million Hong Kong residents. The database documented every diagnosis made by registered clinicians based on the International Classification of Diseases, Ninth Revision, Clinical Modification (ICD-9-CM) with accurate timestamps. Primary care (general outpatient clinic) records were coded according to the International Classification of Primary Care (ICPC) system. Death data were drawn from the city’s death registry, with causes of death coded according to the International Classification of Diseases, Tenth Revision, Clinical Modification (ICD-10-CM).

The date of the recorded antipsychotic drug initiation was used as the index date of the cohort. For the ascertainment of baseline characteristics and inclusion/exclusion criteria, we retrospectively collected chronic disease records (Table S1) within three years prior to the index date and medication usage history (Table S2) within one year. Individuals identified as having multimorbidity were followed up from the index date until the occurrence of the outcomes of interest (described in related sections below), death, or the end of data availability (August 31, 2022), whichever came first. This right censoring allowed the calculation of MACE-free periods from the index date to one of these observation endpoints.

#### Inclusion and exclusion criteria

Records from January 1, 2001, to August 31, 2022, were examined and patients with a record of at least two listed chronic diseases (Table S1) within three years before the index date, i.e., antipsychotic initiation (British National Formulary (B.N.F.) 4.2.1 and 4.2.2, except lithium, were included. Patients who first used antipsychotics before January 1, 2004; had incorrect/ missing records (e.g., death time occurring before the diagnosis date); were not aged 18-65 on the index date; or had MACE before were excluded. As earliest relevant information was only available from 2001, we designated 2001-2003 as the wash-out period for the execution of the exclusion criteria.

#### Outcomes, predictors, and data preparation

MACE was adopted as the study outcome of interest and defined as a composite binary indicator of stroke (ICD-9-CM codes 430 - 438), acute myocardial infarction (AMI) (ICD-9 code 410), or cardiovascular-related death (CV death, ICD-10-CM codes I00 - I02, I05 - I16, I1A, I20 - I28, I30 - I52, I5A, I60 - I89, I95 - I99). Patients with any of these records were considered to have MACE.

Baseline characteristics collected for analyses as independent variables included age, sex, chronic disease history, specific antipsychotic agent initiated, and one-year medication history (Table S2). Diagnostic records were identified by ICD-9 or ICPC codes (Table S1).

We performed random splitting to generate training and validation datasets at a 7:3 ratio stratified by index date. We set 1-year, 3-year, and 5-year time points after the index date as interesting time points in post-hoc model performance diagnostics. The validation dataset was only used for validating machine learning models.

#### Risk group characterization

Based on the wide range of predictors, we employed CISTree to risk-classify MACE within the training dataset only. Renowned for its unbiased recursive partitioning, CISTree is less prone to overfitting compared to conventional decision tree models (Methods S3).^30^

#### Development, validation and interpretation of prediction models

We utilized the Boruta algorithm, configured with 200 iterations, for variable selection. The algorithm evaluates the importance of each feature by comparing it to randomized shadow features, employs statistical testing to rigorously assess feature importance, and iteratively removes irrelevant and redundant variables. This approach minimizes the risk of confounding or misestimation arising from correlated variables, thereby enhancing the model's performance and reliability.^31^ We then trained ten machine learning models—Cox regression model (Cox), generalized linear models with least absolute shrinkage and selection operator regression (LASSO),^32^ Generalized linear models with elastic net regularization (Elastic net),^32^ CISTree,^30^ random survival forest (RSF),^33^ conditional random forest (Cforest),^34^ survival gradient boosting machine (GBM),^35^ survival neural networks (Deepsurv),^36^ extreme gradient boosting survival learner (XGBoost),^37^ and likelihood-based boosting survival Cox model (Coxboost)^38^—using 5-fold cross-validation for grid hyperparameter searching (Methods S3). (modelling and variable selection details are in Table S3) The performance of each model was assessed internally in the training cohort using the “concordance statistics survival measure (survival C-index)” during each validation iteration.^39^

All prediction algorithms were employed to develop and tune models in the training set and then we validated their performance in the validation set. The models’ performance was compared in three aspects—discrimination, calibration, and clinical value which will be reflected by receiver operating characteristic (ROC) curves, calibration plots, and DCA plots.^40^

Advancements in machine learning interpretation have enabled us to explain these “black box” models.^41^ We created time-dependent feature importance plots, partial dependence plots, and Shapley Additive explanations (SHAP) plots for all variables and for specific individual in the training set.^38^^,^^41^^,^^42^^,^^43^

### Quantification and statistical analysis

The main analysis comprised two components: identification of high-risk subgroups for MACE and prediction of MACE risk. Prior to the analysis, the dataset was split into training and validation sets. In the first section, only the training set was used to develop the CISTree model for identifying high-risk MACE subgroups. In the second part, we trained 10 machine-learning models on the training set and evaluated their performance on the validation set. ROC, calibration curve, and decision curve analysis were used in models’ comparison. Model interpretation and deployment of the web-based application were based on the model with the best performance in the validation set. In high-risk of MACE subgroup identification, permutation tests are applied to determine splits.^44^

Sensitivity analyses on all prior diagnoses and on initial exposure to haloperidol, were performed for both risk-group characterization and the prediction model (see Methods S4).

#### Software and algorithms

We conducted all analyses using *R* (version 4·4·0), employing the “*partykit*” (version 4·4·0) package to establish the CISTree model, “*Boruta*” (version 9.0.0) for variable selection, “*mlr3proba*” (version 0.8.6) to build ten machine learning models, “*survex*” (version 1.2.0) for model explanations, and the “*shiny*” (version 1.12.1) package to construct the prediction platform. All continuous baseline variables were skewed and are therefore summarised as medians with interquartile ranges. Incidence rates with 95% confidence intervals were calculated in R. Additional measurement and computational details are publicly available in QS’s Zenodo repository (https://zenodo.org/records/18816892, DOI: https://doi.org/10.5281/zenodo.18816892).

### Additional resources

No additional resources were generated.

## Supplemental information

Document S1. Figures S1–S4, Tables S1–S4, and Methods S1–S4

Table S5. Antipsychotics and other drug use distribution (days)
