# Supplementary material for: Multimorbidity and major adverse cardiovascular events in antipsychotic users: Time-to-event prediction by explainable machine learning
Source: iScience. 2026 Apr 3;29(5):115586. doi: 10.1016/j.isci.2026.115586 (PMC13127323; doi:10.1016/j.isci.2026.115586)
Supplement: Document S1. Figures S1–S4, Tables S1–S4, and Methods S1–S4 [file mmc1.pdf]

iScience, Volume *29*

Supplemental information

Multimorbidity and major adverse cardiovascular events in antipsychotic users: Time-to-event prediction by explainable machine learning

Qi Sun, Wenlong Liu, Cuiling Wei, Yuqi Hu, Lingyue Zhou, Boyan Liu, Rachel Yui Ki Chu, Song Song, Wenxin Tian, Esther Wai Yin Chan, Sherry Kit Wa Chan, Kelvin K.F. Tsoi, Ian Chi Kei Wong, David P.J. Osborn, Daniel Smith, and Francisco Tsz Tsun Lai

Supplementary Table 1. Diagnostic codes used to MACE and operationalize chronic conditions and multimorbidity

Major adverse cardiovascular events (MACE)	International Classification of Diseases, Ninth Revision	The International Classification of Diseases, Tenth Revision, Clinical Modification	International Classification of Primary Care
Acute myocardial infarction (AMI)	410	-	-
Stroke	430,431,432,433,434,436,437,438	-	-
Cardiovascular death	-	I00 - I02, I05 - I16, I1A, I20 - I28, I30 - I52, I5A, I60 - I89, I95 - I99	-
Chronic conditions, and multimorbidity			
Alcohol misuse	265·2,291·1,291·2,291·3,291·5,291·6,291·7,291·8,291·9,303·0,303·9,305·0,357·5,425·5,535·3,571·0,571·1,571·2,571·3,980, V11·3	-	P15
Asthma	493	-	R96
Atrial fibrillation	427·3	-	K78
Cancer, lymphoma	200,201,202,203·0,238·6	-	B72, L71
Cancer, metastatic	196,197,198,199	-	B72, B74, D74, D76, D77, L71, N74, S77, T71, U75, U76, U77, W72, X77, Y78
Cancer, non-metastatic (breast, cervical, colorectal, lung, prostate)	153,154,162,163,174,180,185,230·3,230·4,230·5,230·6,231·2,233·0,233·1,233·4	-	D75, R84, X75, X76, Y77
Chronic kidney disease	583,584,585,586,592,593·9	-	U70, U88, U95
Chronic pain	307·80,307·89,338·0,338·2,338·4,719·41,719·45,719·46,719·47,719·49,720·0,720·2,720·9,721·0,721·1,721·2,721·3,721·4,721·6,721·8,721·9,722,723·0,723·1,723·3,723·4,723·5,723·6,723·7,723·8,723·9,724·0,724·1,724·2,724·3,724·4,724·5,724·6,724·70,724·79,724·8,724·9,729·0,729·1,729·2,729·4,729·5	-	L08, L13, L14, L16, L17, L20, L89, L85, L91, L83, L92, L88, L90, L93, L94, L95, L98, N94, L87
Chronic pulmonary disease	416·8,416·9,490,491,492,494,495,496,497,498,499,500,501,502,503,504,505,506·4,508·1,508·8	-	R95, K82, R79, R81, R96, R98
Chronic viral hepatitis B	70·2,70·3	-	D722, D724
Cirrhosis	571·2,571·5,571·6,456·0,456·1,456·20,456·21,	-	-

	567·0,567·2,567·21,567·29,567·8,567·9,572·2, 572·4,789·5		
Dementia	290,294·1,331·2	-	P70
Depression	296·2,296·3,296·5,300·4,309,311	-	P76
Diabetes	250	-	T89, T90, T901
Epilepsy	345	-	N88
Hypertension	401,402,403,404,405	-	K86, K87
Hypothyroidism	240·9,243,244,246·1,246·8	-	T86
Inflammatory bowel disease	555,556	-	D94
Irritable bowel syndrome	564·1	-	D93
Multiple sclerosis	323,340,341·0,341·9,377·3	-	N86
Parkinson's disease	332	-	N87
Peptic ulcer disease	531·7,531·9,532·7,532·9,533·7,533·9,534·7,53 4·9	-	D86
Peripheral vascular disease	440·2	-	K92
Psoriasis	696·1	-	S91
Rheumatoid arthritis	446·5,710·0,710·1,710·2,710·3,710·4,714·0,71 4·1,714·2,714·8,725	-	L88
Schizophrenia	295	-	P73
Severe constipation	560·1,560·30,560·39,560·9,564·0,569·83,569·89	-	D12
retinal vascular occlusion	362·3	-	-

Supplementary Table 2. British National Formulary codes for the operationalization of medication use

Medications	British National Formulary code
Antianginal drugs	2·6
Beta-adrenoceptor blocking drugs	2·4
Drugs used in diabetes	6·1
Antiplatelet drugs	2·9
Antibacterial drugs	5·1
Corticosteroids (endocrine)	6·3
Diuretics	2·2
Drugs used in hypertension and heart failure	2·5
Antiviral drugs	5·3
Antidepressant drugs	4·3
Drugs for genito-urinary disorders	7·4
Lipid-regulating drugs	2·12
Anti-arrhythmic drugs	2·3
Drugs affecting the immune response	8·2
Anticoagulants and protamine	2·8
Immunoglobulins<br>Antiprotozoal drugs<br>Cough preparations<br>Cytotoxic drugs	14·5<br>5·4<br>3·9·1<br>8·1

Supplementary Table 3. Hyperparameters tuning results and the chosen values

Model	Hyperparameters grid search space	Chosen hyperparameters	Measures (survival C-index)
Cox	-	-	0·779
LASSO	-	-	0·774
Elastic net	Alpha: 0·05 to 0·95	Alpha: 0·55	0·770
CISTree	Alpha: 0·01 to 0·05; Minbucket: 5 to 25	Alpha: 0·01;<br>Minbucket : 20	0·766
RSF	ntree : 200 to 500;<br>mtry : 5 to 27;<br>nodesize : 50 to 200	ntree : 500;<br>mtry : 5;<br>nodesize : 125	0·792
Cforest	ntree : 500 to 800;<br>mtry : 200 to 400	ntree : 500;<br>mtry : 200	0·765
GBM	n·trees : 100 to 500;<br>interaction·depth : 1 to 5;<br>n·minobsinnode : 5 to 21;<br>shrinkage : 0·001 to 0·1	n·trees : 500;<br>interaction·depth : 5;<br>n·minobsinnode : 21;<br>shrinkage : 0·0505	0·798
Deepsurv	num : 1 to 3;<br>nodes : 5 to 13;<br>learning_rate : 0 to 0·1;<br>dropout : 0 to 0·5;<br>weight_decay : 0 to 0·5	num : 1;<br>nodes : 8;<br>learning_rate : 0·011;<br>dropout : 0·167;<br>weight_decay : 0·111	0·780
XGBoost	Nrounds : 100 to 500;<br>max_depth : 1 to 5;<br>eta : 0·0001 to 1	nrounds : 500;<br>max_depth : 2;<br>eta : 0·3334	0·787
Coxboost	Stepno : 1 to 200;<br>Penalty : -10 to 10	Stepno : 42;<br>Penalty : 8·894748	0·772

Supplementary Table 4. Machine learning model's internal validation performance

Tuned models	Survival C-index	Survival calibration score	Right-censored Log Loss
Cox (without hyperparameter tuning)	0·779	0·647	0·607
LASSO (without hyperparameter tuning)	0·774	1·303	0·611
Elastic net	0·774	1·503	0·612
Survival tree	0·790	0·126	0·443
RSF	0·832	4·642	2·088
Cforest	0·895	49·631	0·249
GBM	0·830	777·301	0·912
Deepsurv	0·788	1·641	0·665
XGBoost	0·833	0·645	0·588
CoxBoost	0·779	0·653	0·607

Notes: *: Bold font indicates the best-predicted performance among the ten models. The higher the survival C-index and right-censored Log Loss and the lower the survival calibration score, the better the model performance

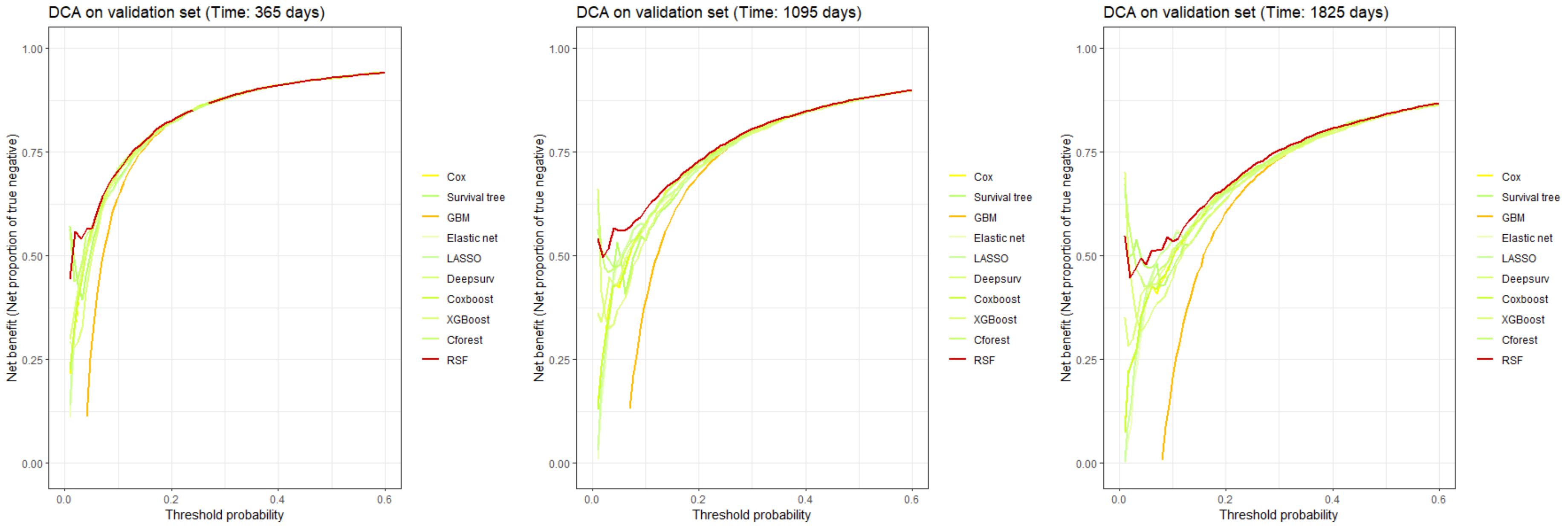

Supplementary Figure 1. DCA plot by using the net proportion of true negative as the net benefit.

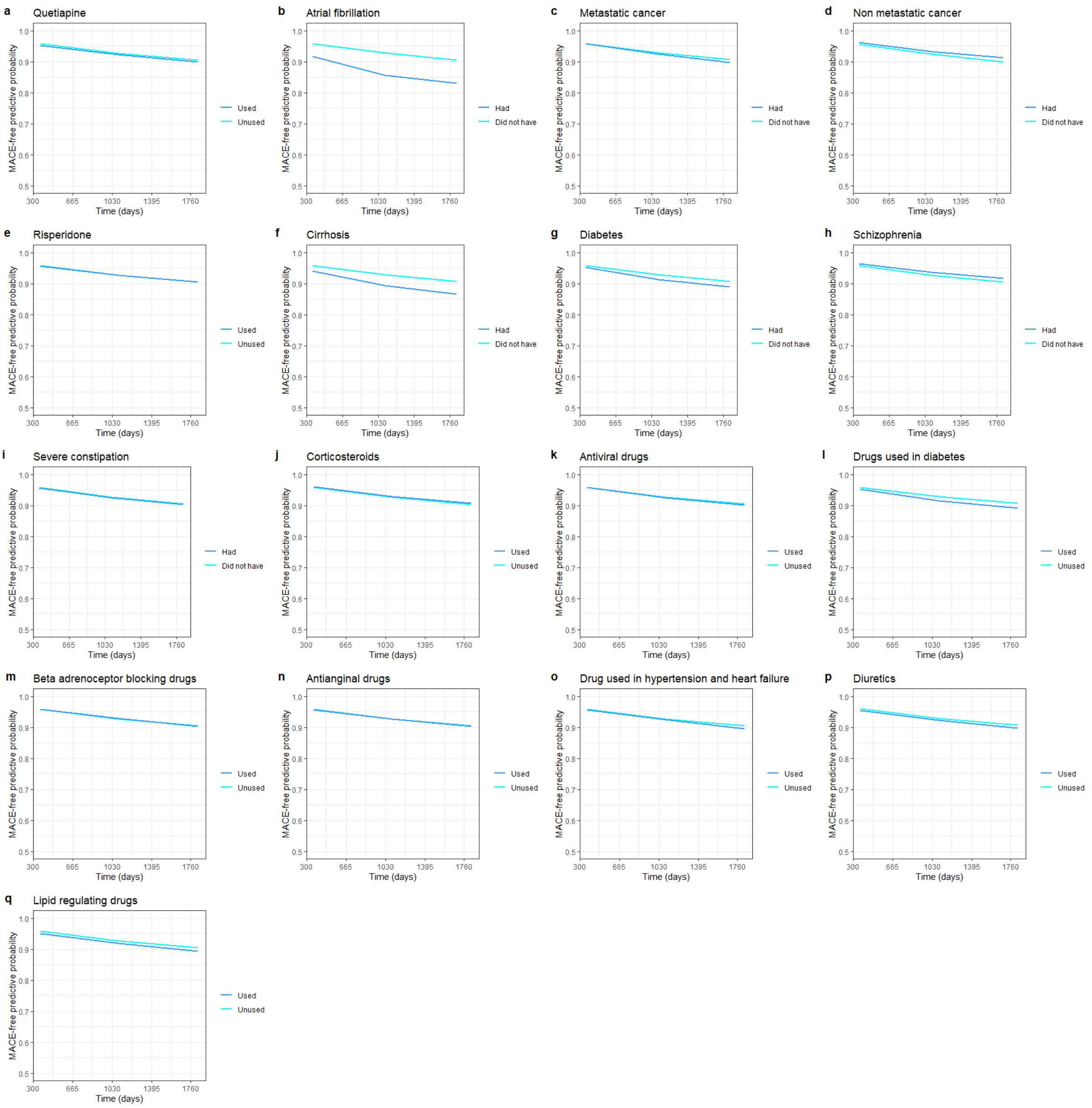

Supplementary Figure 2. Time-dependent partial dependence plots. Data is represented as mean MACE-free predictive probability.

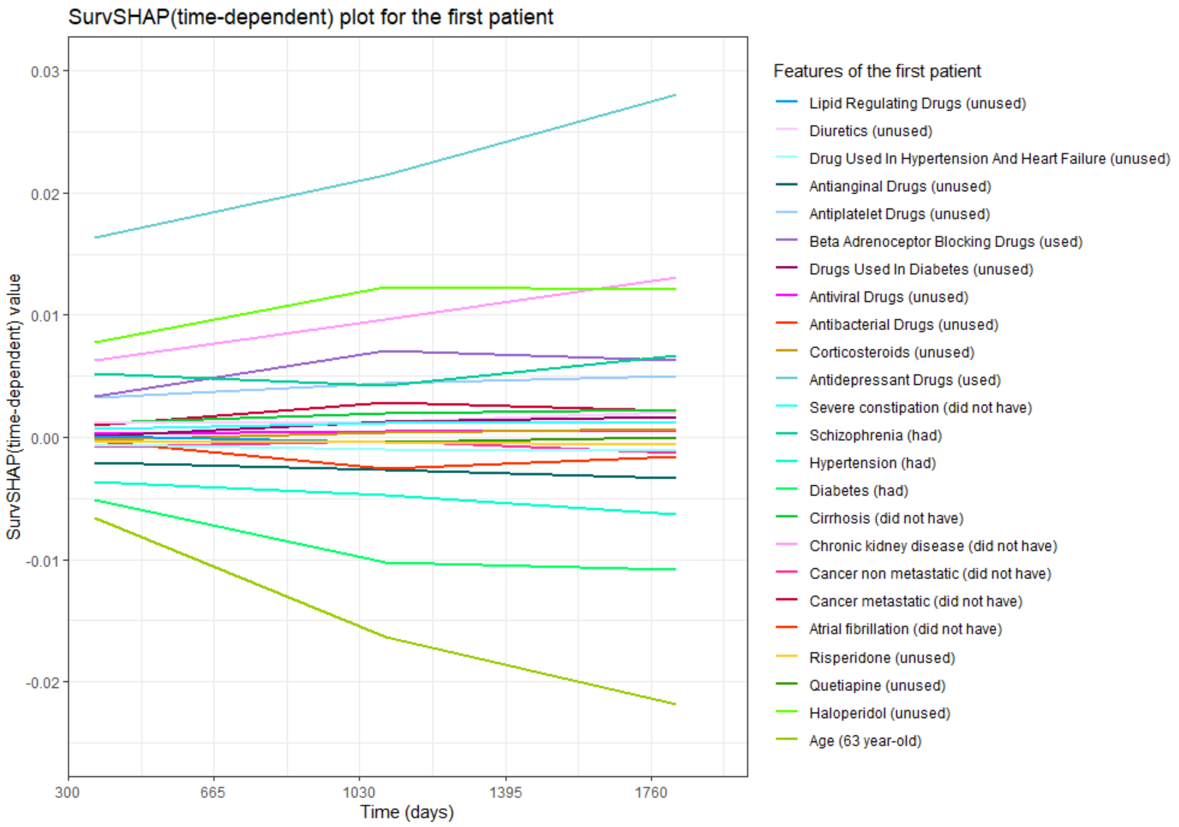

Supplementary Figure 3. SurvSHAP plot of the first patient. The positive SurvSHAP value indicates an increased effect on the survival function, and vice versa

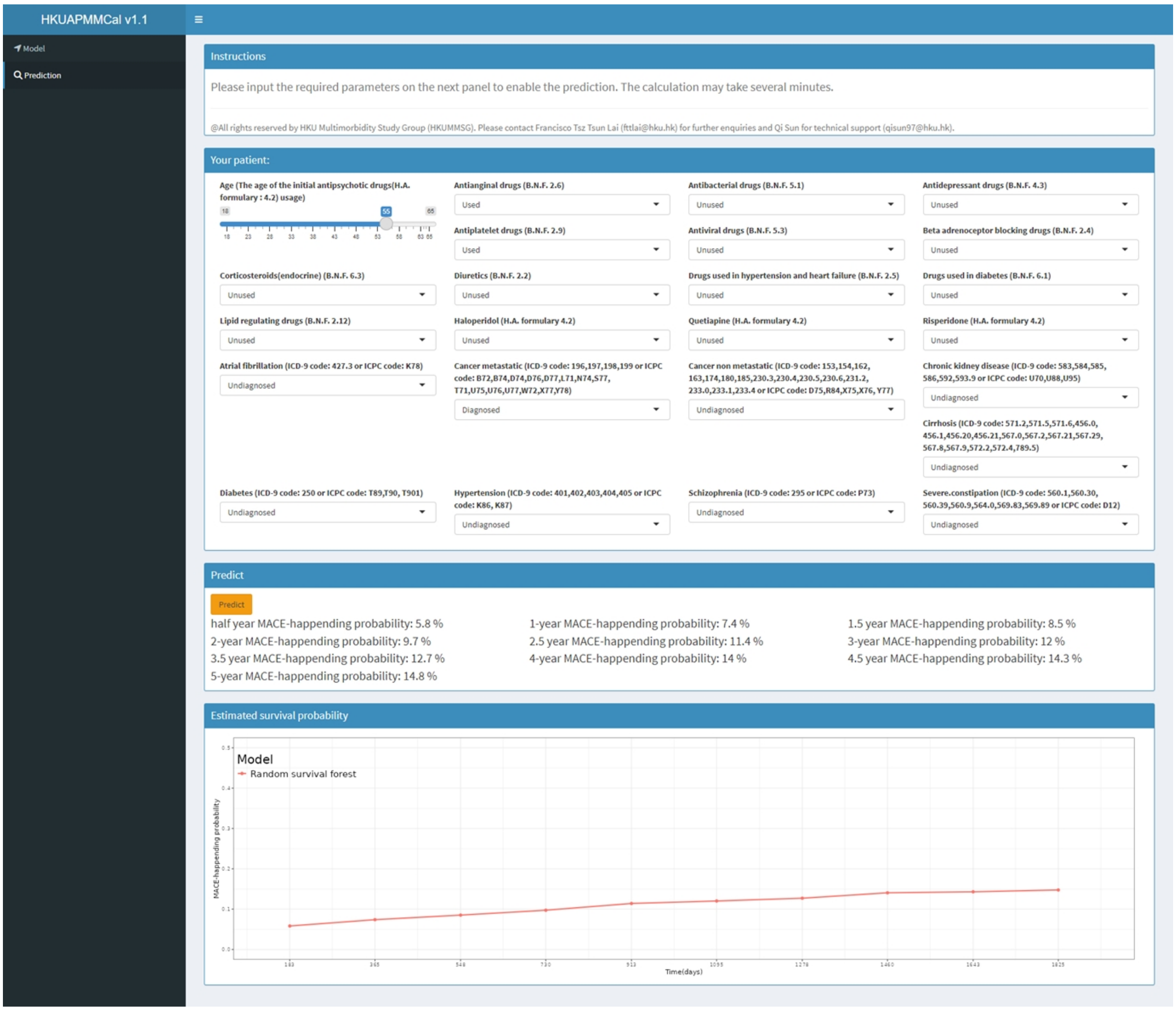

Supplementary Figure 4. HKU Antipsychotics Multimorbidity MACE calculator (HKUAPMMCal)

Methods S1. Decision curve analysis definitions and net benefit formulas

Formulas and variants of net benefit[1]

i) Formulas for net benefit (NB)

NB is calculated as:

$$NB = \frac{TP - odds(T) \times FP}{N},$$

where*TP* is the number of true positives, *FP* the number of false positives, and *N* the sample size. NB divides by *N*, and hence gives the proportion of net true positives.

ii) NB of treat all is

$$NB_{TreatAll} = P - odds(T) \times (1 - P),$$

where*P* is the disease prevalence.

iii) The net proportion of true negatives (i.e. avoided false positives) equals

$$\frac{NB - NB_{TreatAll}}{odds(T)}.$$

Methods S2. External validation protocol for the JMDC claims database

Identifying High-Risk Profiles and Predicting Major Adverse Cardiovascular Events Among Antipsychotic Users with Multimorbidity in Japan: External Validation in the JMDC Claims Database

Rationale

Adults initiating antipsychotics, particularly those with multimorbidity, face an elevated risk of major adverse cardiovascular events (MACE). In prior work using the Hong Kong Hospital Authority database, high-risk subgroups were identified using a Conditional Inference Survival Tree and a machine learning time-to-event model achieved strong discrimination at one, three, and five years among adults aged 18 to 65 years. The critical next step is to assess whether this model transports beyond its development setting without redevelopment or localisation. Japan's JMDC Claims Database provides a nationwide, predominantly working-age population with longitudinal inpatient, outpatient, and dispensing claims since 2005. Although JMDC incompletely captures out-of-hospital mortality and lacks cause-of-death information, it offers high-fidelity capture of inpatient acute myocardial infarction, stroke, and medication exposure. This study is designed as a focused external validation of the Hong Kong–derived MACE prediction model in JMDC. We will preserve the original model's structure and predictor set, apply only the minimal operational harmonisation required to compute model inputs and outcomes, and quantify discrimination, calibration, and clinical utility overall and within prespecified subgroups. By doing so, we aim to generate rigorous evidence on cross-system transportability

Study design and setting

We will conduct a retrospective cohort study in the JMDC Claims Database. The source population comprises insured employees and dependents with longitudinal linkage across care settings. The accrual window will provisionally span 2010 to 2024, with final dates confirmed after data completeness checks. Eligible individuals are adults aged 18 to 65 years who initiate any antipsychotic during the accrual period, defined by the first dispensing date, with at least 365 consecutive days of prior enrollment for baseline ascertainment. New use will be enforced by excluding individuals with any antipsychotic dispensing in the three years before index. Multimorbidity will be defined as the presence of at least two chronic conditions during lookback, constructed to match, as closely as feasible, the predictor constructs required

by the original model. To mirror the development cohort and avoid conditioning on prior outcomes, we will exclude those with a recorded MACE during lookback. Records with implausible or missing essential variables or insufficient lookback will be excluded. Follow-up will begin at index and continue until the first MACE event, disenrollment, recorded death where available, or one, three, and five years, whichever occurs first.

The primary outcome is a composite MACE adapted for JMDC's observable events: hospitalisation for acute myocardial infarction or stroke, and in-hospital cardiovascular death when this can be reliably inferred from discharge disposition in the context of a primary cardiovascular diagnosis or relevant procedure.

Predictors will be operationalised to match the original Hong Kong model's inputs, including demographics, chronic conditions, and medication histories. Hong Kong coding systems will be mapped to JMDC's ICD-10 and NHI drug codes using established crosswalks. Where direct mappings are unavailable, we will define the closest feasible proxies that preserve the intended constructs and document the rationale. If the original model provides regression coefficients and a baseline hazard, we will compute linear predictors and survival probabilities at one, three, and five years. If the model is a machine learning model such as a random survival forest, we will apply the published model artefact or a validated scoring implementation; if only a points-based risk score is available, we will compute the score accordingly. For any model inputs not captured in JMDC, we will follow the original publication's guidance for neutral defaults or missingness handling and perform sensitivity analyses to characterise the impact.

We will describe the JMDC cohort and compare baseline characteristics with the Hong Kong development cohort using standardized mean differences to contextualise case-mix differences. External validation will quantify discrimination using time-dependent ROC curves and area under the curve at one, three, and five years, along with Harrell's C-index. Calibration will be evaluated through calibration-in-the-large, calibration slope, flexible calibration curves, and Brier scores at each time horizon. Clinical utility will be assessed via decision curve analysis across clinically relevant thresholds. All phenotype algorithms, predictor mappings, and any departures from the original specifications will be fully documented. Analyses will be conducted within JMDC's secure environment under data use agreements, with only aggregate, de-identified outputs exported. Ethical approval will be obtained in accordance with institutional requirements for secondary analysis of de-identified claims data.

Prespecified hypothesis

We hypothesise that the Hong Kong–derived MACE prediction model will demonstrate acceptable discrimination and calibration when applied without redevelopment to antipsychotic initiators with multimorbidity aged 18 to 65 years in JMDC at one, three, and five years.

Limitations

Differences in coding granularity and practice patterns may necessitate proxy mappings for certain predictors, introducing construct drift that could affect calibration. Health examination variables present in subsets of JMDC may not be uniformly available and will not be included in the primary validation, potentially limiting comparability if such variables contributed to the original model. Residual confounding and surveillance biases linked to healthcare utilisation intensity may influence observed event capture. Finally, the external validation focuses on working-age insured populations and may not generalise to older adults or uninsured groups.

Planned interpretation and transportability considerations

This study offers a focused and transparent test of cross-system transportability by applying the Hong Kong–derived MACE prediction model, without redevelopment, to a large, independent Japanese claims database. By preserving the model's structure and predictor set and limiting adaptations to the minimum necessary for operationalisation, we isolate performance differences attributable to case mix, coding, and outcome capture rather than to model refitting. Evaluating discrimination, calibration, and clinical utility at clinically relevant horizons will clarify the model's capacity to stratify cardiovascular risk among antipsychotic initiators with multimorbidity in Japan. If simple recalibration suffices to correct miscalibration, the model could be deployed pragmatically in Japanese settings while retaining the original predictor effects; if not, the results will delineate specific gaps and inform priorities for model adaptation or redevelopment in future work. Beyond immediate applicability, the findings will contribute to a broader evidence base on the transportability of cardiovascular risk prediction across Asian healthcare systems, highlighting the conditions under which externally developed models can perform reliably without localisation.

Methods S3. Additional methodological details for model development and tuning

Conditional inference survival tree development and hyperparameter tuning details

CISTree is a non-parametric, tree-based embedding model that utilizes a conditional inference procedure. Fundamentally, the algorithm generates weights for each learning sample and subsequently tests for independence between the covariates and the outcome for case weights. If a relationship is present, the predictor with the most evident link with the outcome (indicated by the lowest P-value, compared using the "Bonferroni – Monte Carlo adjusted P-value") is selected.[2,3] We set the minimum number of cases in any terminal nodes to 1240 (three times the number of participants needed to observe a case, and we extend to 30 times due to enough cases for time-to-event analysis).[4]

Boruta variable selection algorithm introduction

Briefly, it initially randomly adds a shuffled copy of all factors, then returns features importance scores from a random forest classifier consecutively, where it automatically detects and erases a real feature which is relatively less important than its shadow one simultaneously.[5] The process stops when every feature is confirmed or rejected or it reaches 200 iterations. Only confirmed features were included in the training and validation sets in subsequent steps.

Machine learning algorisms development

1. Cox regression model (Cox)

Cox is a semi-parametric statistic model for survival regression or prediction. We choose all selected variables by the Boruta algorism to establish Cox model. (without hyperparameters tuning)

2. Generalized linear models with least absolute shrinkage and selection operator regression (LASSO)

LASSO is a regularization Cox model with the parameter - alpha equals to 1. We choose all selected variables by the Boruta algorism to establish LASSO model. [6]

3. Generalized linear models with elastic net regularization (Elastic net)

Elastic net is a regularization Cox model with the hyperparameter – alpha from 0 to 1, which should be tuned to find the suitable value. We choose all selected variables by the Boruta algorism to establish elastic net model.[6]

4. Survival trees

Survival tree is an extension of classification and regression tree (CART), however the outcome is the time-to-MACE status, at each time point whether one will occur MACE. We choose all selected variables by the Boruta algorism to establish Survival tree model. Hyperparameters include complexity parameter (cp) and minbucket, the smallest number of cases in a partitionable node.[2]

5. Random survival forest (RSF)

RSF is an extension of random forest, a bagging machine learning method. The splitting rule is "Log-rank splitting", which is to maximize the difference of two log-rank statistics in daughter nodes. We choose all selected variables by the Boruta algorism to establish RSF model. The hyperparameters of RSF include the number of trees to build the forest, the number of variables randomly selected for splitting at each node, and minimum size of terminal nodes.[7]

6. Conditional random forest (Cforest)

Cforest is an extension of conditional inference tree in survival framework. It is also a bagging algorism. Unlike RSF, the Cforest choose the variable with the strongest association to survival outcome to a splitting variable, and apply permutation test to find the best splitting value. We choose all selected variables by the Boruta algorism to establish Cforest model. The hyperparameters of Cforest include the number of trees to build the Cforest and the number of variables randomly selected for splitting at each node.[8]

7. Survival gradient boosting machine (GBM)

Survival gradient boosting machine is an extension of gradient boosting machine, a boosting algorism. Unlike forest methods, averaging all base learners results then making a final decision, GBM and other boosting methods will focus on the poor samples in each iteration, and finally get one result. Here, the base learner is survival tree. We choose all selected variables by the Boruta algorism to establish GBM model. The hyperparameters of GBM include the number of trees to build the GBM, the number of splits on a tree, learning rate, and minimum size of terminal nodes, which contributes to the stop of GBM.[9]

8. Survival neural networks (Deepsurv)

Deepsurv is a deep learning Cox-based model, which replaces the linear part in Cox by non-linear relationship studied by neural network framework. We choose all selected variables by the Boruta algorism to establish Deepsurv model and it will automatically find the suitable weights between predictors and survival functions. The hyperparameters include the number of nodes in each hidden layer, the number of hidden layers, learning rate, dropout layer, and weights decay.[10]

9. Extreme gradient boosting survival learner (XGBoost)

XGBoost is another boosting method besides GBM, which including regularization and provide tree pruning to avoid overfitting. And the base learner is Cox. Globally, it should perform better than GBM on test dataset. We choose all selected variables by the Boruta algorism to establish XGBoost model. The hyperparameters include the number of boosting iterations, the maximum depth, and the learning rate.[11]

10. Likelihood-based boosting survival Cox model (Coxboost)

Compared to gradient boosting methods, it uses the offset-based boosting method of Tutz and Binder to estimate the Cox model. And in each iteration, the offset of the partial likelihood estimate penalized by the previous boosting step is used to obtain an update of a single parameter, i.e., a covariate. We choose all selected variables by the Boruta algorism to establish Coxboost model. The hyperparameters include the number of boosting steps and the penalty value for each updated parameter in each iteration.[12]

Methods S4. Sensitivity analyses for robustness to haloperidol imbalance and prior diagnoses

i. Sensitivity analysis for antipsychotics imbalance – splitting original data into no-haloperidol and with-haloperidol groups

Identification of MACE risk group

No-haloperidol

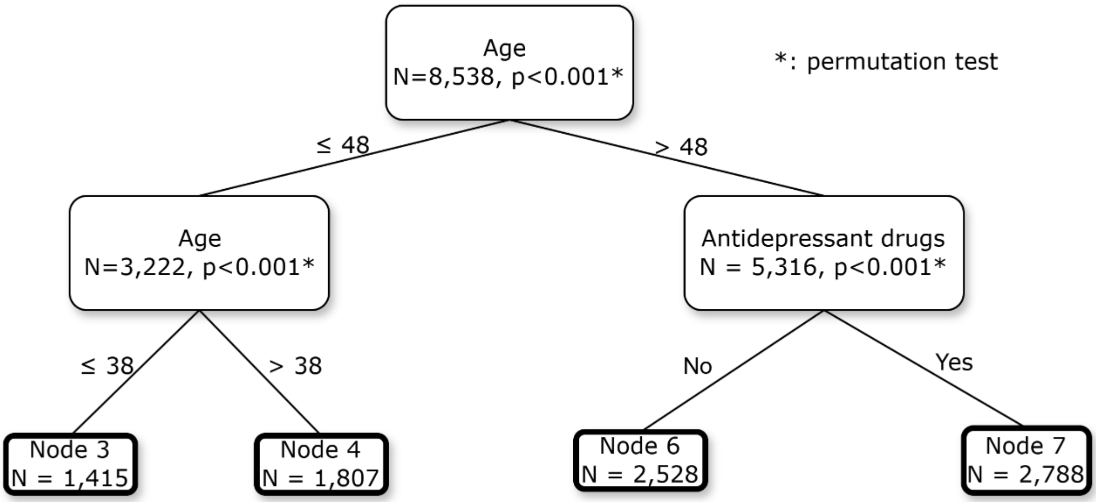

	Node 3	Node 4	Node 6	Node 7	Total
MACE case number	22	90	291	202	605
MACE person years	11640.040	13650.108	9536.482	18401.019	53227.649
MACE incidence rate (IR)	1.890	6.593	30.514	10.978	11.366
MACE IR 95% Lower Limit	1.184	5.302	27.109	9.516	10.478
MACE IR 95% Upper Limit	2.862	8.104	34.229	12.600	12.309
MI case number	5	18	76	52	151
MI person years	12409.693	15816.013	19977.516	21494.882	69698.104
MI incidence rate (IR)	0.403	1.138	3.804	2.419	2.166
MI IR 95% Lower Limit	0.131	0.675	2.997	1.807	1.835
MI IR 95% Upper Limit	0.940	1.799	4.762	3.172	2.541
Stroke case number	16	69	179	137	401
Stroke person years	12328.957	15600.909	19337.436	21027.157	68294.459
Stroke incidence rate (IR)	1.298	4.423	9.257	6.515	5.872
Stroke IR 95% Lower Limit	0.742	3.441	7.950	5.470	5.311
Stroke IR 95% Upper Limit	2.107	5.597	10.717	7.702	6.475
CV-death case number	2	9	71	30	112
CV-death person years	12413.435	15870.461	19815.817	21547.911	69647.624
CV-death incidence rate (IR)	0.161	0.567	3.583	1.392	1.608
CV-death IR 95% Lower Limit	0.020	0.259	2.798	0.939	1.324
CV-death IR 95% Upper Limit	0.582	1.077	4.519	1.988	1.935

Data is represented as point estimated incidence rates and 95% confidence interval.

With haloperidol

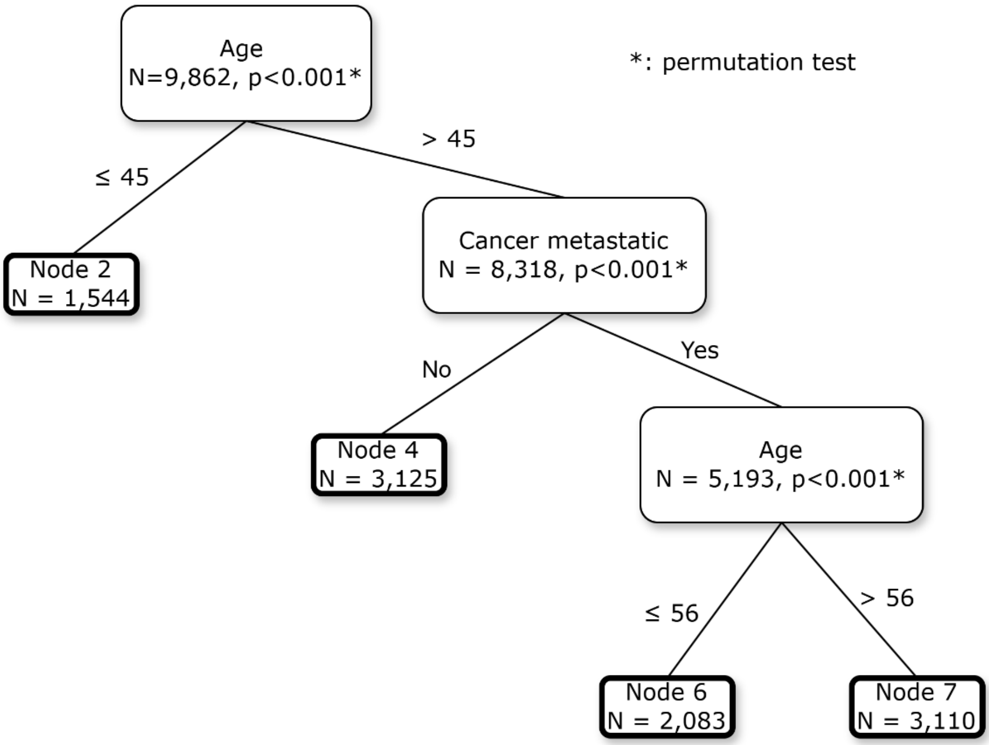

	Node 2	Node 4	Node 6	Node 7	Total
MACE case number	56	516	23	60	655
MACE person years	8678.948	13706.872	1073.952	1318.072	24777.844
MACE incidence rate (IR)	6.452	37.645	21.416	45.521	26.435
MACE IR 95% Lower Limit	4.874	34.467	13.576	34.737	24.449
MACE IR 95% Upper Limit	8.379	41.038	32.135	58.595	28.539
MI case number	12	140	1	13	166
MI person years	16563.378	29702.230	21301.267	26562.494	94129.369
MI incidence rate (IR)	0.724	4.713	0.047	0.489	1.764
MI IR 95% Lower Limit	0.374	3.965	0.001	0.261	1.505
MI IR 95% Upper Limit	1.266	5.562	0.262	0.837	2.053
Stroke case number	40	305	19	41	405
Stroke person years	16386.616	28288.689	21136.768	26359.892	92171.965
Stroke incidence rate (IR)	2.441	10.782	0.899	1.555	4.394
Stroke IR 95% Lower Limit	1.744	9.606	0.541	1.116	3.976
Stroke IR 95% Upper Limit	3.324	12.062	1.404	2.110	4.843
CV-death case number	10	172	4	11	197
CV-death person years	16527.964	19232.592	21296.482	26546.287	93603.325
CV-death incidence rate (IR)	0.605	5.884	0.188	0.414	2.105
CV-death IR 95% Lower Limit	0.290	5.037	0.051	0.207	1.821
CV-death IR 95% Upper Limit	1.113	6.832	0.481	0.741	2.420

Data is represented as point estimated incidence rates and 95% confidence interval.

RSF performance across subgroups

No-haloperidol

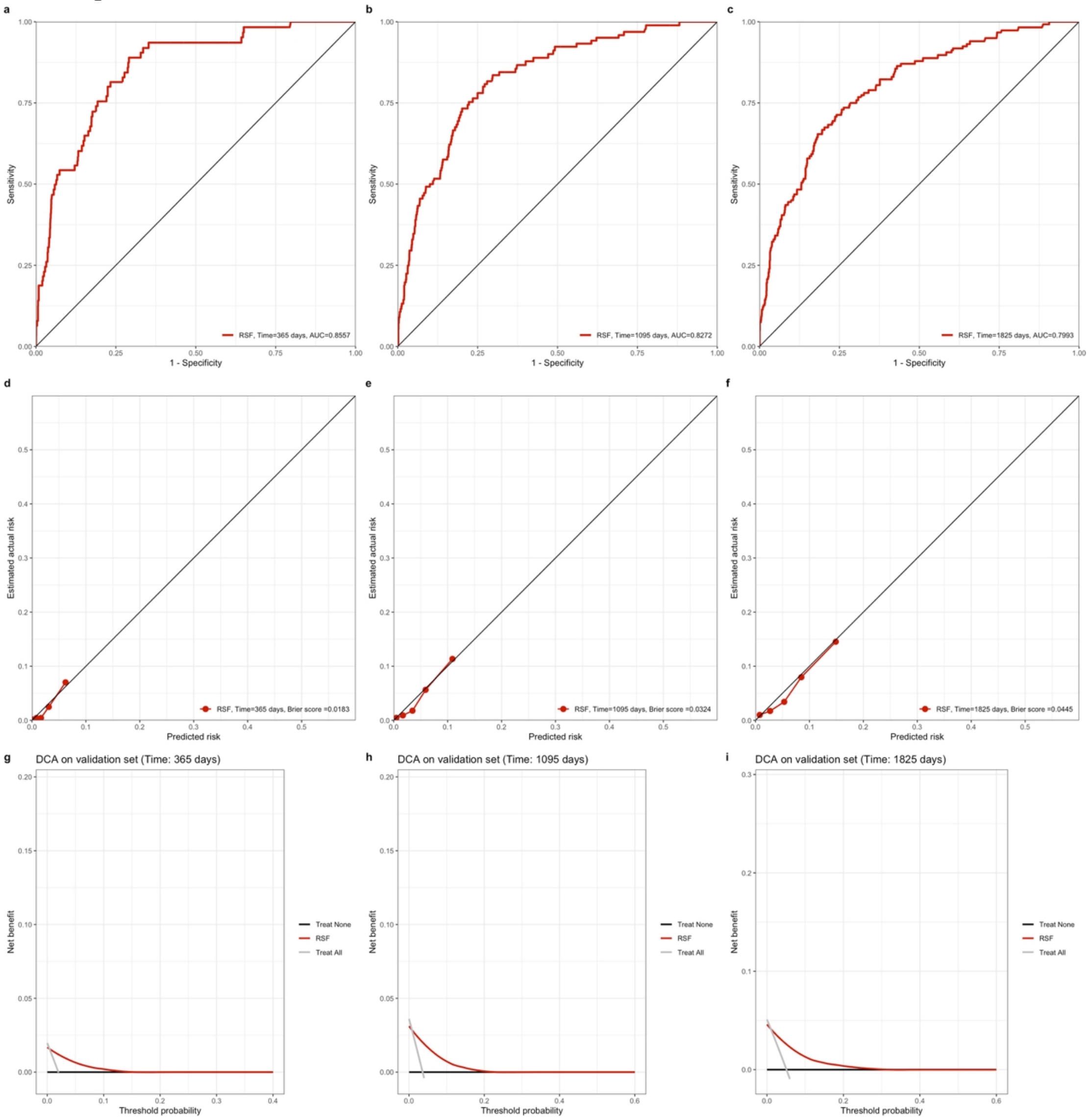

With haloperidol

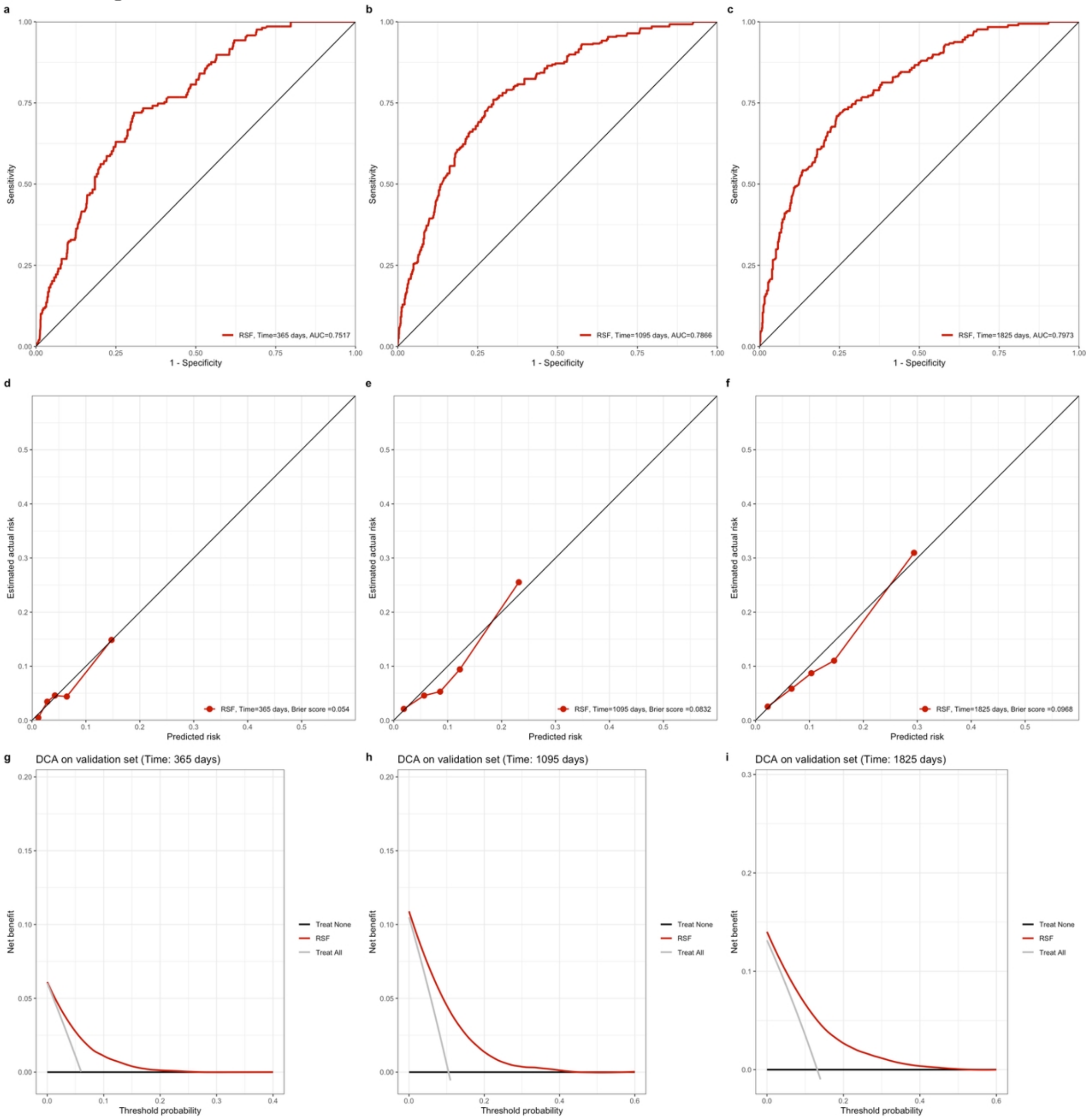

ii. Sensitivity analysis for using all past diagnosis

Identification of MACE risk group

For all patients

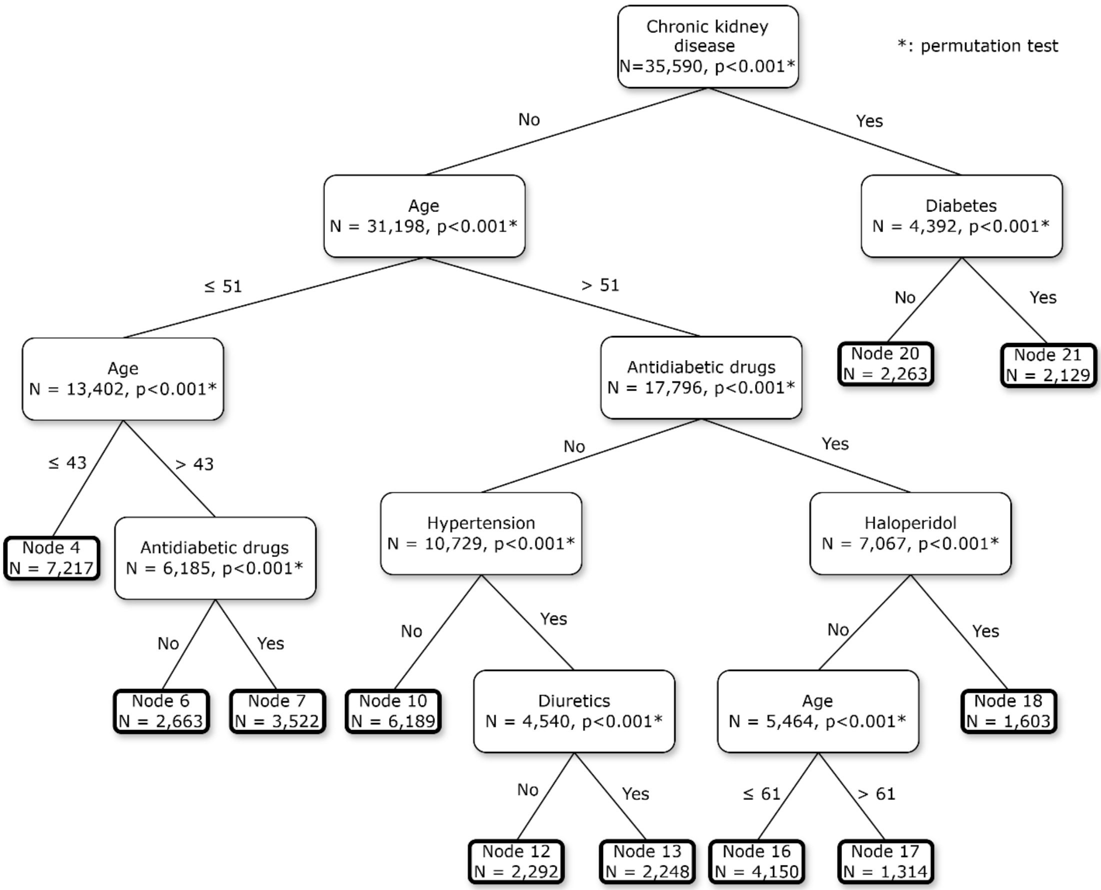

	Node 4	Node 6	Node 7	Node 10	Node 12	Node 13	Node 16	Node 17	Node 18	Node 20	Node 21	Total
MACE case number	147	146	149	300	432	161	216	122	116	283	515	2587
MACE person years	50043.261	10884.298	24289.407	13887.769	9303.144	6495.368	26046.797	6791.704	5124.624	8541.612	4818.066	166226.050
MACE incidence rate (IR)	2.937	13.414	6.134	21.602	46.436	24.787	8.293	17.963	22.636	33.132	106.889	15.563
MACE IR 95% Lower Limit	2.482	11.326	5.189	19.226	42.160	21.106	7.224	14.917	18.704	29.384	97.856	14.969
MACE IR 95% Upper Limit	3.453	15.775	7.202	24.190	51.028	28.925	9.476	21.448	27.149	37.226	116.533	16.175
MI case number	31	21	29	69	73	60	52	25	20	82	225	687
MI person years	58082.053	23933.225	28679.201	51237.967	18730.046	15737.840	28272.323	8083.153	15173.112	16614.894	14251.102	278794.916
MI incidence rate (IR)	0.534	0.877	1.011	1.347	3.897	3.812	1.829	3.093	1.318	4.935	15.788	2.464
MI IR 95% Lower Limit	0.363	0.543	0.677	1.048	3.055	2.909	1.374	2.002	0.805	3.925	13.793	2.283
MI IR 95% Upper Limit	0.758	1.341	1.452	1.704	4.900	4.907	2.412	4.566	2.036	6.126	17.992	2.656
Stroke case number	115	115	117	193	332	80	147	87	83	156	207	1632
Stroke person years	57665.510	23395.129	28212.508	50390.565	17303.483	15558.634	27915.054	7858.298	14760.373	16066.892	14199.334	273325.78
Stroke incidence rate (IR)	1.994	4.916	4.147	3.830	19.187	5.142	5.266	11.071	5.623	9.709	14.578	5.971
Stroke IR 95% Lower Limit	1.646	4.058	3.430	3.309	17.178	4.077	4.449	8.867	4.479	8.246	12.660	5.685
Stroke IR 95% Upper Limit	2.394	5.900	4.970	4.410	21.366	6.399	6.189	13.656	6.971	11.358	16.705	6.268
CV-death case number	8	20	11	64	84	38	32	17	23	105	227	629
CV-death person years	58120.136	23911.390	28741.807	51174.067	18525.052	15861.543	28381.581	8136.489	15111.216	16295.903	13997.809	278256.993
CV-death incidence rate (IR)	0.138	0.836	0.383	1.251	4.534	2.396	1.127	2.089	1.522	6.443	16.217	2.261
CV-death IR 95% Lower Limit	0.059	0.511	0.191	0.963	3.617	1.695	0.771	1.217	0.965	5.270	14.176	2.087
CV-death IR 95% Upper Limit	0.271	1.292	0.685	1.597	5.614	3.288	1.592	3.345	2.284	7.800	18.469	2.444

Data is represented as point estimated incidence rates and 95% confidence interval.

No-haloperidol

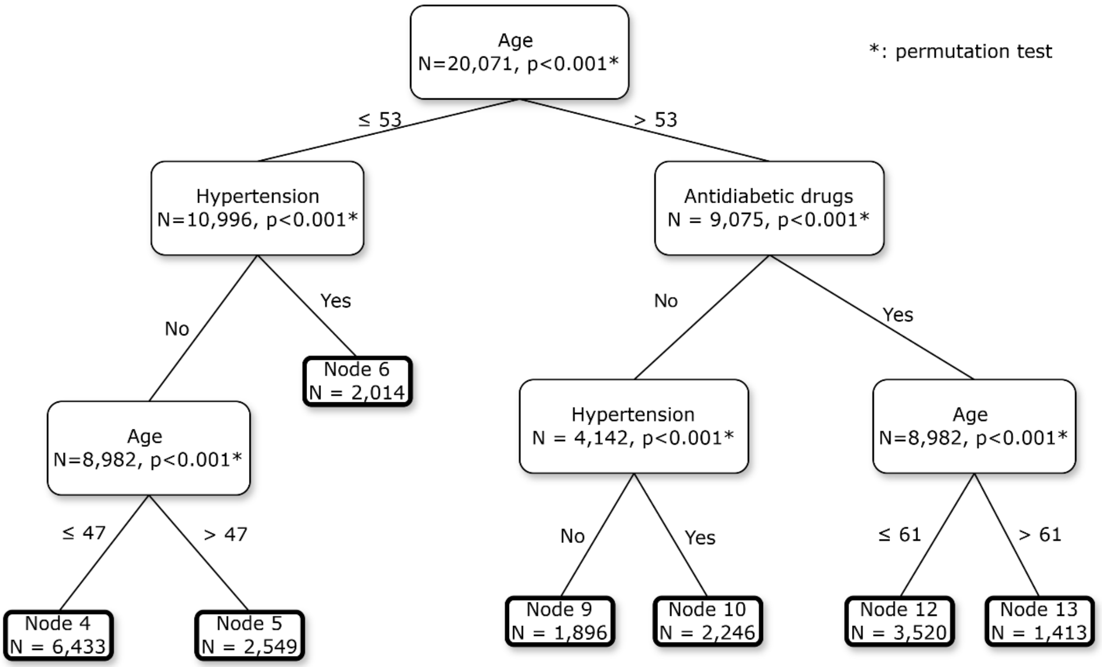

	Node 4	Node 5	Node 6	Node 9	Node 10	Node 12	Node 13	Total
MACE case number	126	125	151	144	399	198	128	1271
MACE person years	44992.345	16388.557	12617.97	6938.176	8966.312	21204.663	7183.640	118291.663
MACE incidence rate (IR)	2.800	7.627	11.967	20.755	44.500	9.338	17.818	10.745
MACE IR 95% Lower Limit	2.333	6.349	10.134	17.503	40.240	8.082	14.865	10.162
MACE IR 95% Upper Limit	3.334	9.088	14.035	24.435	49.088	10.733	21.186	11.352
MI case number	21	22	42	39	89	49	27	289
MI person years	47976.918	19534.689	14317.419	13529.293	15142.743	23289.782	8576.033	142366.877
MI incidence rate (IR)	0.438	1.126	2.933	2.883	5.877	2.104	3.148	2.030
MI IR 95% Lower Limit	0.271	0.706	2.114	2.050	4.720	1.556	2.075	1.803
MI IR 95% Upper Limit	0.669	1.705	3.965	3.941	7.233	2.782	4.581	2.278
Stroke case number	108	97	98	89	263	134	91	880
Stroke person years	47627.122	19090.983	14083.137	13217.644	14238.897	22982.493	8339.534	139579.81
Stroke incidence rate (IR)	2.268	5.081	6.959	6.733	18.471	5.831	10.912	6.305
Stroke IR 95% Lower Limit	1.860	4.120	5.649	5.407	16.305	4.885	8.786	5.895
Stroke IR 95% Upper Limit	2.738	6.198	8.480	8.286	20.843	6.905	13.397	6.735
CV-death case number	6	11	23	34	96	33	18	221
CV-death person years	48037.845	19528.786	14395.894	13537.803	14946.993	23396.325	8633.720	142477.366
CV-death incidence rate (IR)	0.125	0.563	1.598	2.511	6.423	1.410	2.085	1.551
CV-death IR 95% Lower Limit	0.046	0.281	1.013	1.739	5.202	0.971	1.236	1.353
CV-death IR 95% Upper Limit	0.272	1.008	2.397	3.510	7.843	1.981	3.295	1.770

Data is represented as point estimated incidence rates and 95% confidence interval.

With haloperidol

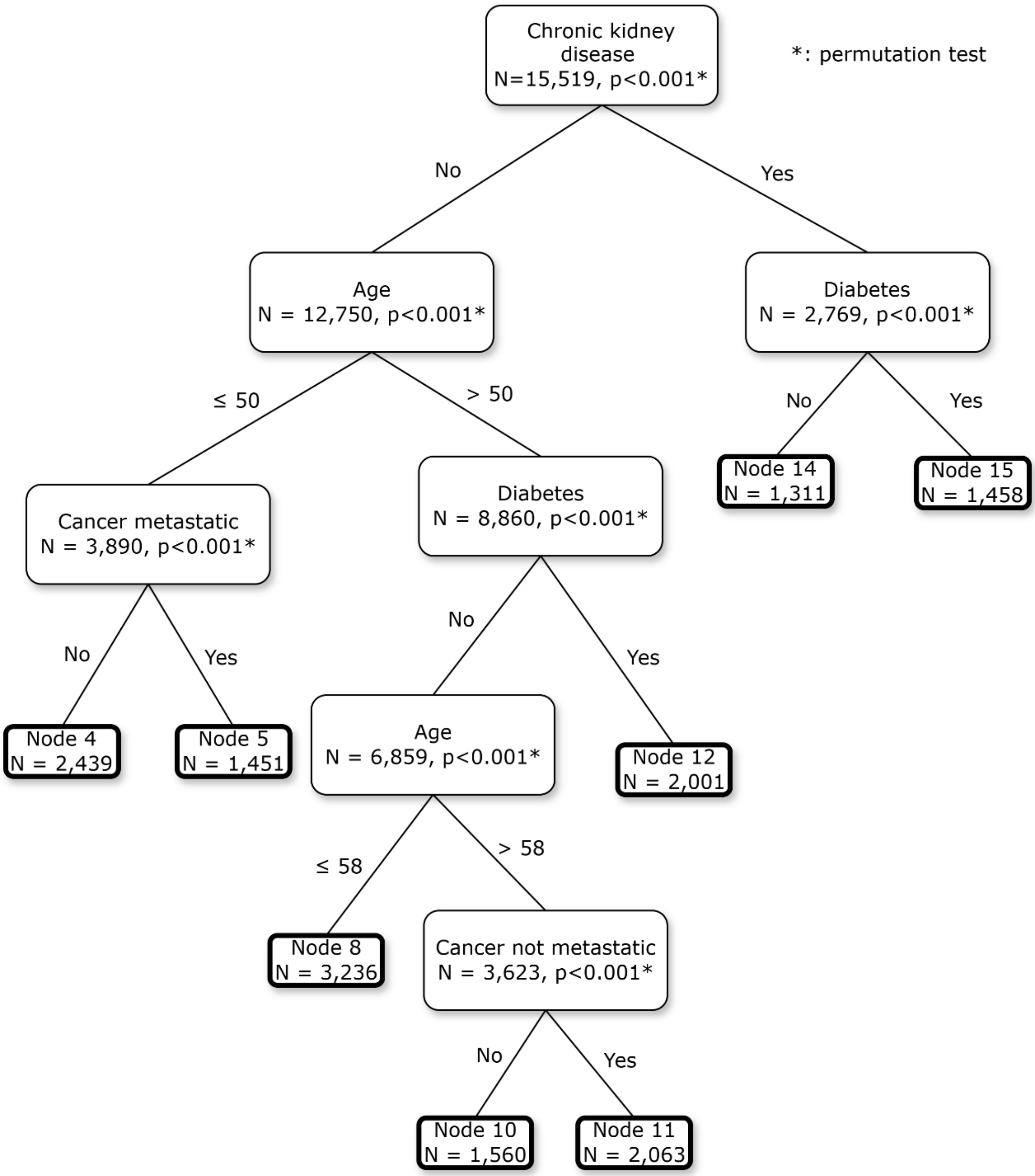

	Node 4	Node 5	Node 8	Node 10	Node 11	Node 12	Node 14	Node 15	Total
MACE case number	125	12	150	162	42	245	194	386	1316
MACE person years	20295.685	1067.659	8153.118	4853.667	1425.318	5820.019	3699.685	2619.236	47934.387
MACE incidence rate (IR)	6.159	11.240	18.398	33.377	29.467	42.096	52.437	147.371	27.454
MACE IR 95% Lower Limit	5.127	5.808	15.571	28.435	21.237	36.989	45.317	133.034	25.991
MACE IR 95% Upper Limit	7.338	19.633	21.589	39.931	39.831	47.711	60.358	162.832	28.979
MI case number	22	3	33	35	8	64	57	176	398
MI person years	23920.952	15071.722	30732.268	12626.186	17582.463	16542.414	9968.238	9983.796	136428.039
MI incidence rate (IR)	0.920	0.199	1.074	2.772	0.455	3.869	5.718	17.629	2.917
MI IR 95% Lower Limit	0.576	0.041	0.739	1.931	0.196	2.979	4.331	15.12	2.638
MI IR 95% Upper Limit	1.392	0.582	1.508	3.855	0.897	4.940	7.409	20.434	3.218
Stroke case number	92	9	105	111	29	151	103	152	752
Stroke person years	23496.846	15025.948	30223.311	12103.419	17435.656	15878.923	9577.369	10004.498	133745.970
Stroke incidence rate (IR)	3.915	0.599	3.474	9.171	1.663	9.509	10.757	15.193	5.623
Stroke IR 95% Lower Limit	3.156	0.274	2.842	7.544	1.114	8.053	8.778	12.874	5.228
Stroke IR 95% Upper Limit	4.802	1.137	4.206	11.044	2.389	11.153	13.043	17.810	6.039
CV-death case number	18	2	22	36	8	66	79	177	408
CV-death person years	23874.375	15080.285	30797.748	12536.78	17558.937	16452.578	9663.742	9815.182	135779.627
CV-death incidence rate (IR)	0.754	0.133	0.714	2.872	0.456	4.012	8.175	18.033	3.005
CV-death IR 95% Lower Limit	0.447	0.016	0.448	2.011	0.197	3.103	6.472	15.474	2.720
CV-death IR 95% Upper Limit	1.192	0.479	1.082	3.975	0.898	5.104	10.188	20.894	3.311

Data is represented as point estimated incidence rates and 95% confidence interval.

RSF performance across subgroups

All patients

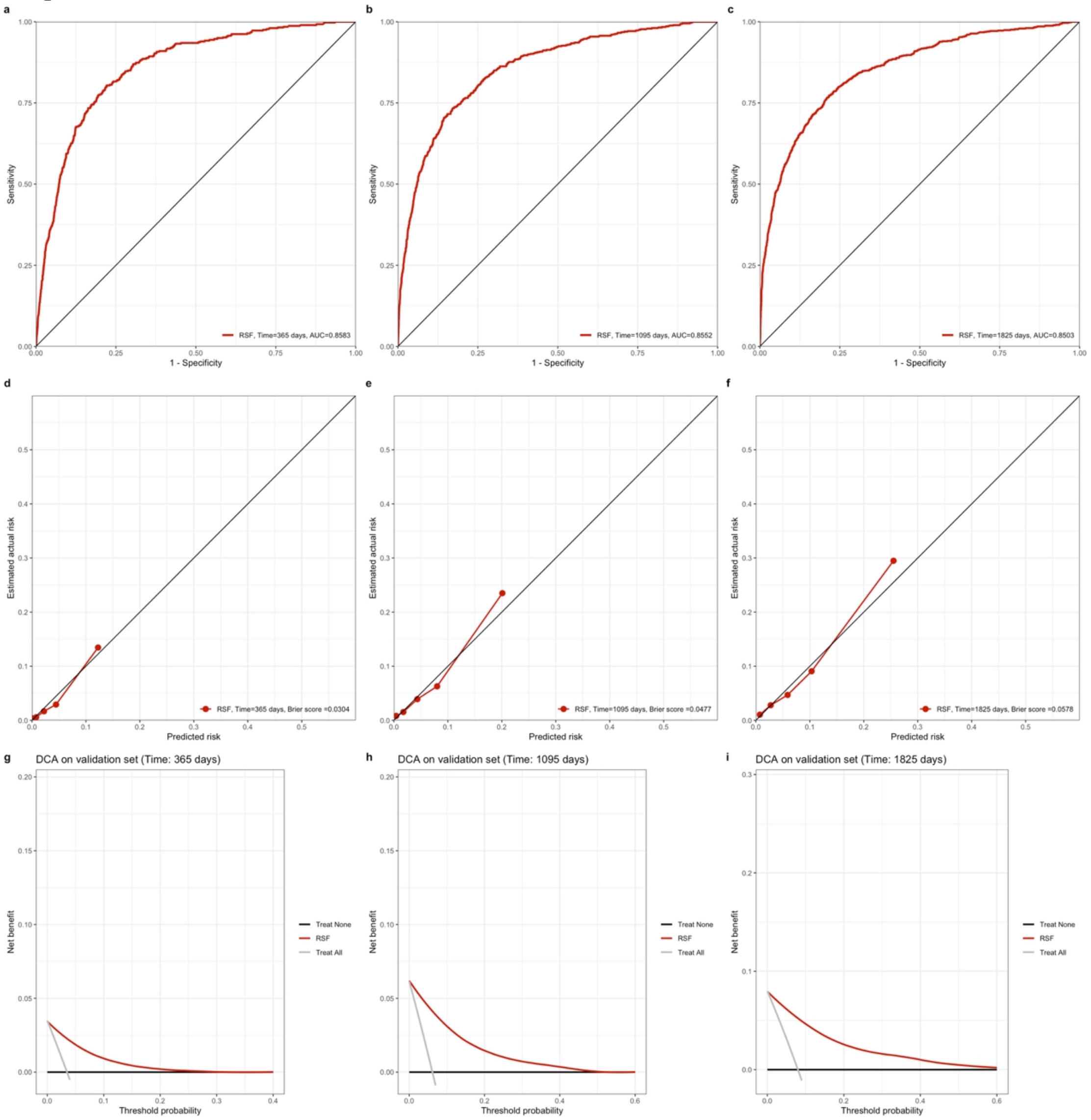

No-haloperidol

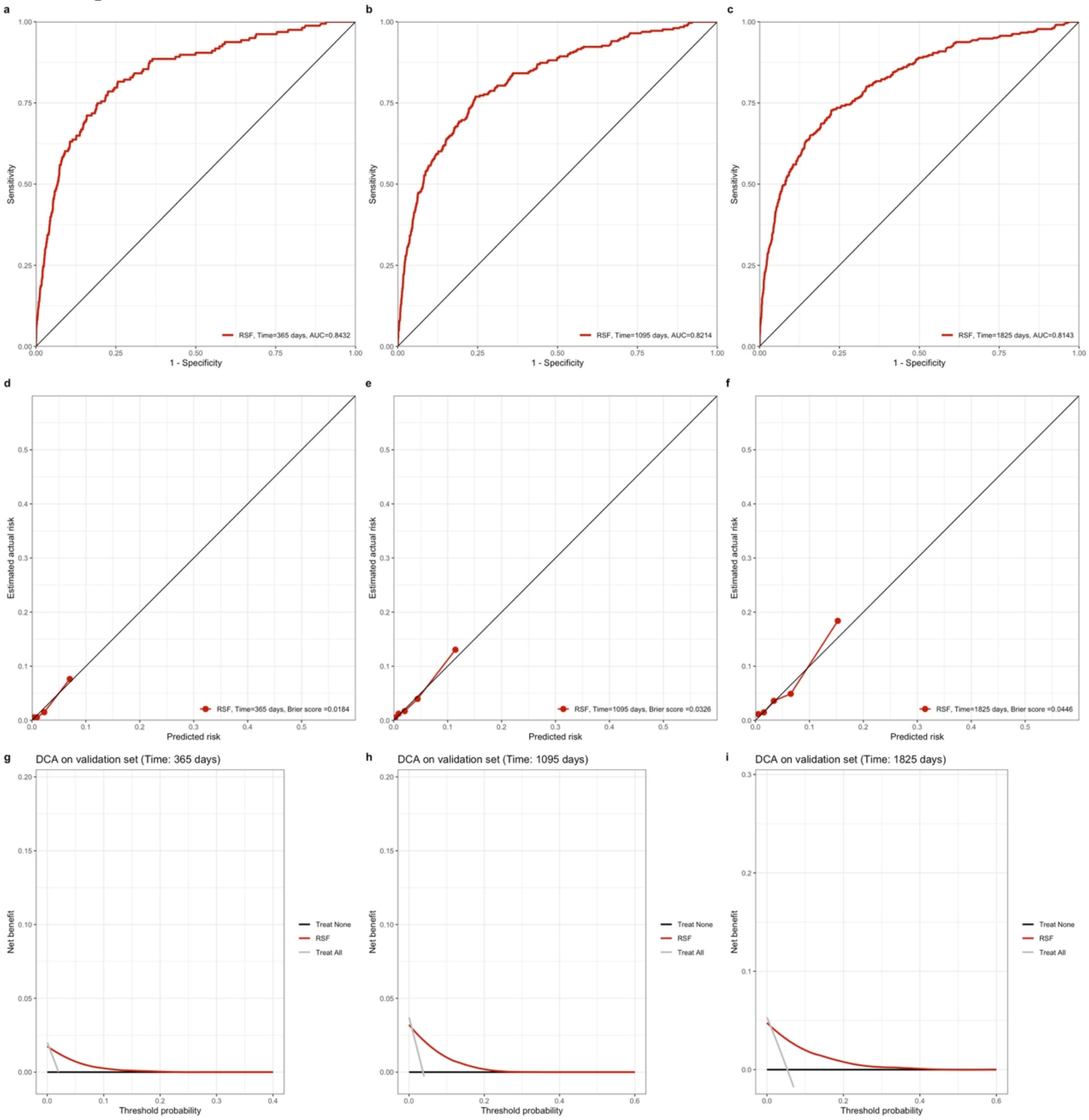

With haloperidol

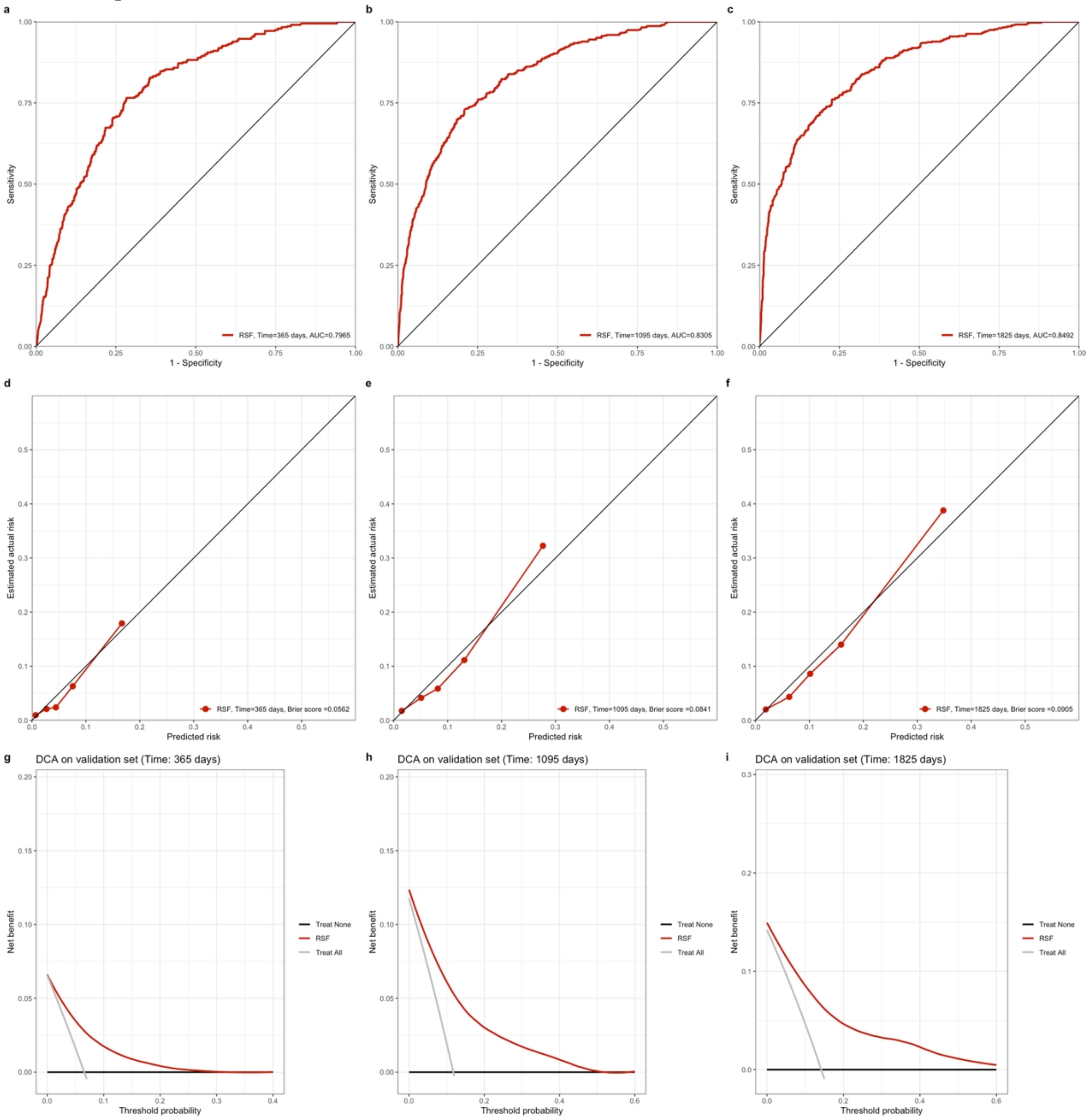

Supplemental References

1. Van Calster B, Wynants L, Verbeek JFM, et al. Reporting and Interpreting Decision Curve Analysis: A Guide for Investigators. *Eur Urol.* 2018;74(6):796-804.
2. Hothorn T, Hornik K, Zeileis A. ctree: Conditional inference trees. *The comprehensive R archive network.* 2015;8:1-34.
3. Strobl C, Boulesteix A-L, Augustin T. Unbiased split selection for classification trees based on the Gini Index. *Computational Statistics & Data Analysis.* 2007;52(1):483-501.
4. Onakpoya IJ. Rare adverse events in clinical trials: understanding the rule of three. *BMJ Evidence-Based Medicine.* 2018;23(1):6-6.
5. Kursa MB, Rudnicki WR. Feature Selection with the Boruta Package. *Journal of Statistical Software.* 2010;36(11):1 - 13.
6. Friedman JH, Hastie T, Tibshirani R. Regularization Paths for Generalized Linear Models via Coordinate Descent. *Journal of Statistical Software.* 2010;33(1):1 - 22.
7. Ishwaran H, Kogalur UB, Blackstone EH, Lauer MS. Random survival forests. 2008.
8. Hothorn T, Zeileis A, Wien WW. partykit: A Toolbox for Recursive Partytioning.
9. Friedman JH. Stochastic gradient boosting. *Computational Statistics & Data Analysis.* 2002;38(4):367-378.
10. Katzman JL, Shaham U, Cloninger A, Bates J, Jiang T, Kluger Y. DeepSurv: personalized treatment recommender system using a Cox proportional hazards deep neural network. *BMC Med Res Methodol.* 2018;18(1):24.
11. Chen T, Guestrin C. XGBoost: A Scalable Tree Boosting System. Proceedings of the 22nd ACM SIGKDD International Conference on Knowledge Discovery and Data Mining; 2016; San Francisco, California, USA.
12. Binder H, Allignol A, Schumacher M, Beyersmann J. Boosting for high-dimensional time-to-event data with competing risks. *Bioinformatics.* 2009;25(7):890-896.
